# mTOR inhibition enhances the antitumor efficacy of pan-RAF-MEK blockade by inhibiting the ATF4-MTHFD2 pathway

**DOI:** 10.1038/s41419-026-08836-5

**Published:** 2026-05-06

**Authors:** Feiyang Cai, Fan Huang, Christophe Goncalves, Harinee Srikannan, Natascha Gagnon, Elizabeth M. Guettler, Léa Mukeba-Harchies, Jennifer Maxwell, Jie Su, Krikor Bijian, Fabrice Journe, April A. N. Rose, Alexandre Orthwein, Sonia Victoria del Rincón, Wilson H. Miller

**Affiliations:** 1 Division of Clinical and Translational Research, McGill University, Montreal, QC Canada; 2 Lady Davis Institute, McGill University, Montreal, QC Canada; 3 Cancer Research Unit, Department of Human Biology and Toxicology, University of Mons, Mons, Belgium; 4 Department of Radiation Oncology, Winship Cancer Institute, Emory University, Atlanta, GA USA; 5 Department of Oncology, McGill University, Montreal, QC Canada

**Keywords:** Melanoma, Melanoma, Stress signalling

## Abstract

BRAF V600 inhibitors are clinically approved for the treatment of BRAF^V600^-mutant melanoma in combination with a MEK inhibitor, but are ineffective in other melanoma subtypes. Moreover, pan-RAF inhibitors, such as belvarafenib, when combined with MEK inhibitors (cobimetinib), have promising but limited efficacy in non-*BRAF*-mutant melanomas. Here, we report that the mTOR inhibitor sapanisertib improves the efficacy of combined belvarafenib and cobimetinib therapy in *NRAS*, *NF1*, and *KIT*-mutant melanomas. Mechanistically, sapanisertib combined with belvarafenib and cobimetinib suppressed ATF4 expression and its target gene MTHFD2 while inducing DNA damage, revealing a previously underappreciated role of the ATF4-MTHFD2 axis in DNA damage repair and drug response. Human and murine models resistant to combined belvarafenib and cobimetinib exhibited elevated levels of ATF4 and MTHFD2 and were sensitive to sapanisertib. This study provides promising treatment opportunities for patients with non-*BRAF*-mutant melanomas, or those who relapse following belvarafenib and cobimetinib combination therapy.

## Introduction

Melanoma is one of the most aggressive skin cancers, being highly metastatic [1]. Cutaneous melanoma can be classified into four genomic subtypes based on the mutation status of *BRAF*, *NRAS*, *NF1*, and a fourth subgroup termed triple wild-type [2]. The RAS-RAF-MEK-ERK pathway is an essential signal transduction cascade in all melanoma subtypes [3, 4]. FDA-approved *BRAF V600* inhibitors [4, 5], when combined with MEK inhibitors, cause rapid regression of *BRAF*-mutant melanomas, which account for around 50% melanoma cases. However, these drugs are unable to inhibit both protomers of the RAF dimer and paradoxically activate MAPK signaling in non-*BRAF V600* mutant melanoma [6–9]. *NRAS*-mutant melanomas exhibit greater aggressiveness and are associated with a poorer prognosis in comparison to *BRAF*-mutant melanomas [10]. Currently, there is no FDA-approved targeted therapy for patients with *NRAS*-mutant, *NF1*-mutant, and triple wild-type melanomas, making them “hard-to-treat” subtypes and highlighting the critical need for novel therapies for such patients.

A novel pan-RAF inhibitor, belvarafenib, has shown promising pre-clinical results in *NRAS*-mutant melanoma [11]. Combined belvarafenib and cobimetinib was trialed in *NRAS*-mutant melanoma patients (NCT03284502), with an overall response rate (ORR) of 38.5% [12], but resistance remains a major challenge, as observed in nearly all patients treated with targeted therapy [12, 13]. In addition to MAPK signaling, driver mutations in melanoma often simultaneously activate the PI3K-AKT-mTOR pathway, which is an essential signaling cascade driving MAPK inhibitor resistance in melanoma [14–17]. Importantly, most studies in melanoma examining the role of mTOR in the context of MAPK pathway-targeted therapies have focused largely on the *BRAF*-mutant tumors. As a result, the contribution of mTOR signaling to therapeutic response and resistance in hard-to-treat subtypes remains largely unexplored. Furthermore, to cope with stresses from rapid cell division and anti-tumor therapy, cancer cells often activate the integrated stress response (ISR) pathway, governed by activating transcription factor 4 (ATF4) [18]. Mechanistically, mTOR can control ATF4 translation [19], and plays an important role in resistance to BRAF-targeted therapy [20]. Given the essential roles of the mTOR pathway and ISR signaling in therapy tolerance and resistance, we sought to investigate whether blocking mTOR would augment therapeutic responses to combined belvarafenib plus cobimetinib therapy. We chose to use sapanisertib (*a.k.a* INK128), an ATP-competitive catalytic inhibitor of mTOR [21] that has been evaluated in clinical trials to treat a variety of solid tumors (NCT02412722, NCT02197572), showing well-tolerated adverse effects [22, 23]. Unlike first-generation mTOR inhibitors [24], sapanisertib targets both mTORC1 and mTORC2, blocking feedback loops that reactivate AKT, thereby expanding its potential in therapy-resistant tumors and providing a rationale for its use in combination with other anti-cancer agents. Indeed, we found that sapanisertib potentiated the anti-tumor effects of belvarafenib plus cobimetinib both in vitro and in vivo, including in therapy-resistant melanomas. We identified that the ATF4-MTHFD2 axis facilitates DNA repair in belvarafenib plus cobimetinib-treated cells, thus promoting their therapy tolerance. We subsequently found that the therapeutic benefit of belvarafenib + cobimetinib + sapanisertib (referred hitherto as triple therapy) included the ability of mTOR inhibition to rewire MTHFD2-mediated purine synthesis and DNA repair in the belvarafenib plus cobimetinib-treated melanomas. Though toxicity might be anticipated with a triple therapy, our in vitro and in vivo data in models of hard-to-treat melanoma show that there is robust anti-tumor activity without associated toxic side effects, thereby supporting the overall therapeutic benefit of the triple therapy.

## Materials and methods

### Mice

Male C57BL/6N mice (6–8 weeks old) were purchased from Charles River Laboratories. Female nonobese diabetic (NOD)/severe combined immunodeficiency (SCID) mice (6–10 weeks old) were kindly gifted by Dr. Moulay Alaoui-Jamali. All mice were randomized before injection, and the order in which the analysis procedures were performed was done at random. Blinding was not applied as the studies involved specific drug treatment cohorts.

For melanoma cell inoculation, MaNRAS1007 cells were injected into male C57BL/6N mice at 1,000,000 cells/mouse. WM3406 cells were injected into female NOD/SCID mice at 1,000,000 cells/mouse. All melanoma cell lines were freshly prepared in PBS and subcutaneously injected into the right flank of mice. Once palpable tumors were formed, tumors were measured in length (*L*) and width (*W*). Tumor volumes (*V*) were calculated based on the formula *V* = 3.1416/6 × *L* × *W*^2^.

For belvarafenib (5 mg/kg, MedChem Express, HY-109080), cobimetinib (5 mg/kg, ChemieTek CT-G0973), and INK128 (0.5 mg/kg, MedChem Express, HY-13328) treatment, these drugs were dissolved in DMSO and subsequently diluted in PEG400 (Sigma-Aldrich, P3265), TWEEN® 20 (Sigma-Aldrich, P1379), and PBS. Mice were administered every day by gavage, starting when the tumor volume reached approximately 100 mm^3^. For the tumor growth curve, mice were sacrificed when the tumor volume reached approximately 1500 mm^3^. For IHC in Fig. 6, all mice were sacrificed on Day 20 after treatment initiation. The IHC on relapsed tumors were performed when the tumor volume reached 1000 mm^3^.

### Cells and reagents

Sources, culture conditions, and treatment timelines of human melanoma cell lines are listed in Supplementary Table 3. Drug treatments began following cell seeding. For nucleoside supplementation, nucleosides (100X, Sigma-Aldrich, ES-008) with 0.73 g/L cytidine, 0.85 g/L guanosine, 0.73 g/L uridine, 0.8 g/L adenosine, and 0.24 g/L thymidine were added 1:100 in cell culture media. Cells were collected for apoptosis assay and Western blot after 48 h of treatment. All cell lines were authenticated by short tandem repeat analysis and confirmed to be free of mycoplasma contamination.

### Statistics

Sample sizes were determined based on prior experience and similar assays. No statistical test was used to pre-determine the sample sizes. No data was excluded. In vitro data are presented as mean ± SD. In vivo data are presented as mean ± SEM. Prism software (GraphPad) was used to determine the statistical significance of differences. Figure legends specify the statistical analysis used. *P* values are indicated in the figures, and *p* < 0.05 were considered significant.

Additional experimental details are provided in the Supplementary Materials and “Methods” section. Full and uncropped western blots are available in Supplementary Materials.

## Results

### Inhibiting mTOR augments the efficacy of combined belvarafenib and cobimetinib in melanoma cells

**Fig. 1:**
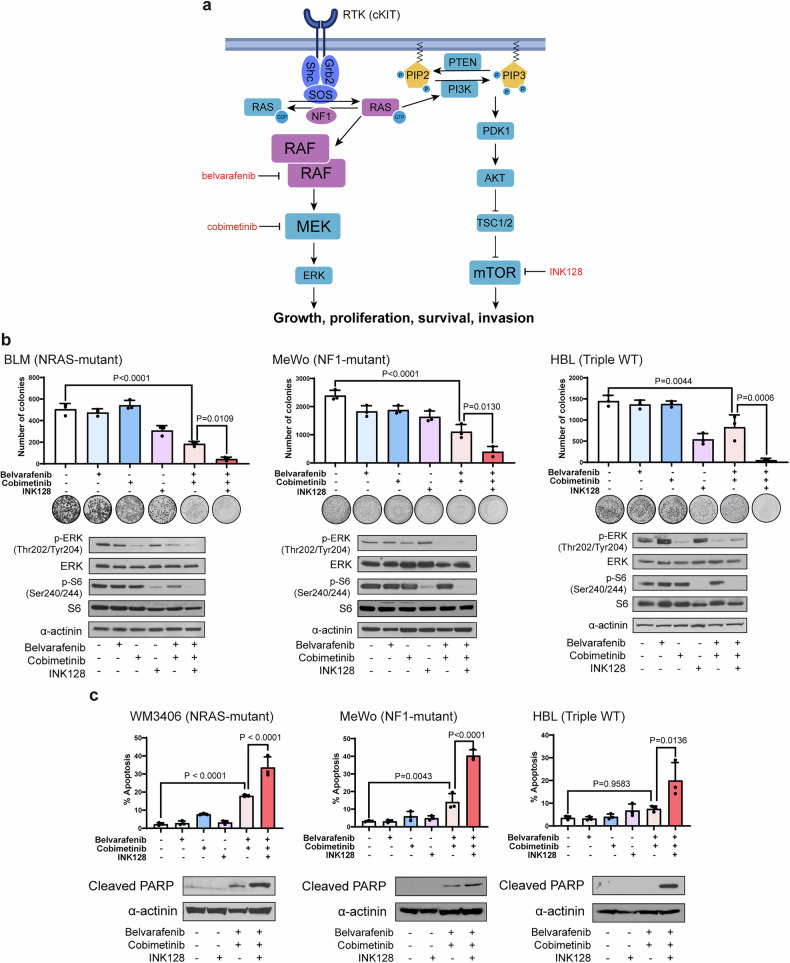
The inhibition of mTOR augments the anti-cancer effects of belvarafenib and cobimetinib combination therapy in melanoma cells. **a** Schematic diagram showing the MAPK signaling and PI3K/AKT/mTOR signaling in melanoma cells. **b** Top: Colony number assays show that mTOR inhibition with INK128 enhances the growth suppressive effect of combined belvarafenib and cobimetinib in human melanoma cells following 10 days of treatment (belvarafenib, 25 nM; cobimetinib, 50 nM; INK128, 25 nM). Bottom: Western blot analysis of the indicated proteins in human melanoma cells following the treatment of belvarafenib, cobimetinib, and INK128 for 2 h (*n* = 3), confirming effective inhibition of MAPK and mTOR signaling. One-way ANOVA. **c** Top: Percentage apoptotic cells as measured by the sum of PI/Annexin V double-positive and Annexin V-positive staining. Bottom: Western blot analysis of the indicated proteins in human melanoma cells. Both assays show that the addition of INK128 increases apoptosis induced by combined belvarafenib and cobimetinib. One-way ANOVA. All data represent mean ± SD.

Oncogenic mutations of *NRAS*, *NF1*, and *KIT* in melanoma drive hyperactivated RAS signaling, which contributes to activated MEK/ERK and PI3K/mTOR signaling [25]. Given the emerging role of the mTOR pathway in melanoma response and resistance to MAPK inhibition [14], we hypothesized that targeting the mTOR pathway could enhance the anti-tumor activity of belvarafenib plus cobimetinib combination therapy in non-*BRAF*-mutant melanoma cells. We thus determined the half-maximal inhibitory concentration (IC_50_) of belvarafenib and cobimetinib in the context of combination therapy using a panel of human melanoma cell lines harboring different genetic mutations (*NRAS*^*Q61R*^-mutant BLM cells, *NRAS*^*Q61K*^-mutant WM3406 and WM3623 cells, *NF1*^Q1336*^-mutant MeWo cells, *c-KIT*^*D820Y*^-mutant HBL cells, and *NRAS*^*G12V*^-mutant YUGOE cells [26]) (Supplementary Fig. 1a). Next, we treated melanoma cell lines with belvarafenib (25 nM), cobimetinib (50 nM), and the mTOR inhibitor sapanisertib (hereafter referred to as INK128 [21], 25 nM) and monitored their impact on colony formation capacity (Fig. 1a, b and Supplementary Fig. 1b, c). As expected, belvarafenib plus cobimetinib significantly decreased colony formation, but some cells persisted, surviving the 10-day treatment (Fig. 1b and Supplementary Fig. 1b). Notably, there were fewer colonies in all four melanoma cell lines treated with the triple therapy (Fig. 1b and Supplementary Fig. 1b). All cell lines were tested to confirm on-target engagement (Fig. 1a, b). We further showed that the triple therapy induced the highest level of apoptosis at 48 h. Interestingly, INK128 did not induce apoptosis on its own, consistent with its anti-cancer effects being mostly cytostatic, rather than cytolytic [21, 27], but significantly increased belvarafenib plus cobimetinib-induced apoptosis in all the melanoma subtypes tested (Fig. 1c and Supplementary Fig. 1c). We recognize that a triple therapy may carry unwanted toxicity; thus, we verified that the triple therapy did not induce apoptosis in two non-malignant melanocyte lines, MelST [28], and Melan-A [29] (Supplementary Fig. 1c).

### Transcriptomics reveals DNA damage and stress response pathways as downregulated in the triple therapy

**Fig. 2:**
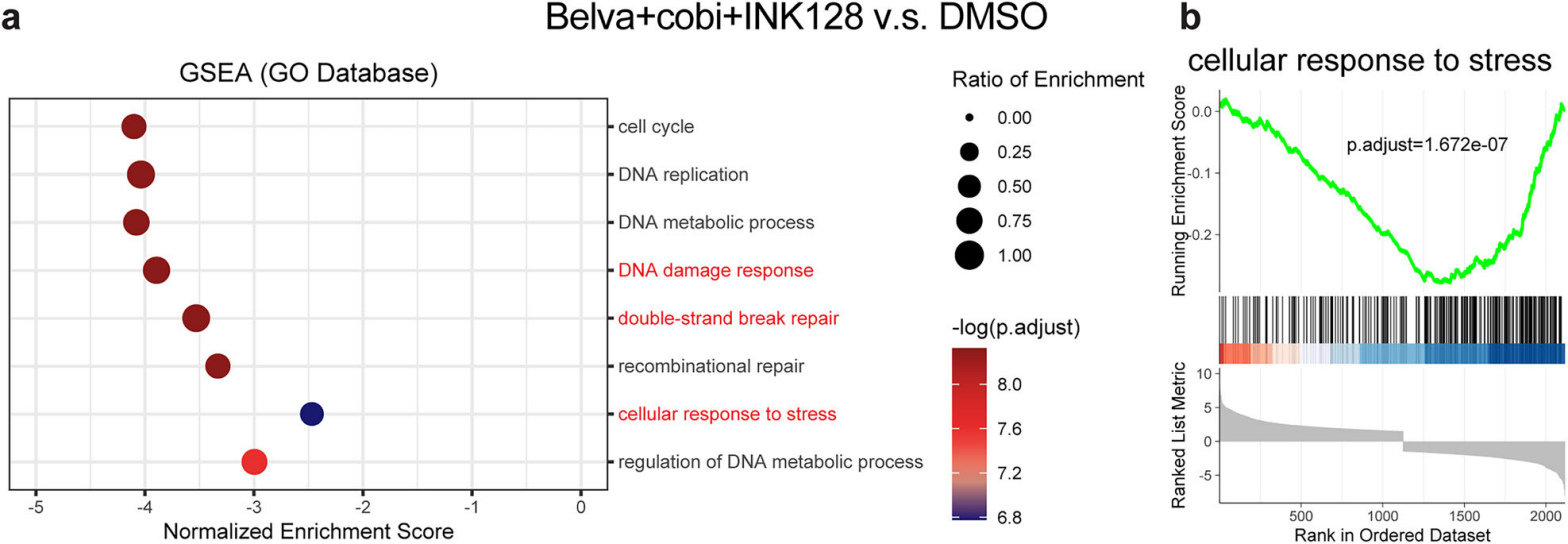
Transcriptomics reveals DNA damage and stress response pathways as downregulated in the triple therapy. **a** Dotplot depicting Gene Ontology (GO) enrichment of differentially expressed genes (DEGs) in triple therapy-treated cells, compared with DMSO treatment. Gene sets that were potentially related to the apoptosis phenotype were colored red, including DNA damage response, double-strand break repair, and cellular response to stress. Fisher test. **b** GSEA for these DEGs in cellular response to stress, indicating the inhibition of cellular stress programs following combined MAPK and mTOR inhibition.

To investigate potential mechanisms through which the triple therapy eliminated melanoma cells, we performed RNA sequencing analysis on the *NRAS*-mutant WM3406 cell line (Supplementary Fig. 2a, b). Gene set enrichment analysis (GSEA) on differentially expressed genes (DEGs) in cells treated with the triple therapy versus the vehicle-treated cells identified the “cellular response to stress” pathway, centered on the ISR signaling, as being downregulated in the triple therapy-treated cells (Fig. 2a, b, Supplementary Fig. 2c–f, and Supplementary Table 1). Such data prompted us to further explore the activation of the ISR, which can be marked by increased expression of its major effector, ATF4, and plays a crucial role in supporting persister cell survival during BRAF-targeted therapy [30].

### The triple therapy induces apoptosis of melanoma cells through the inhibition of ATF4 and the induction of DNA damage

**Fig. 3:**
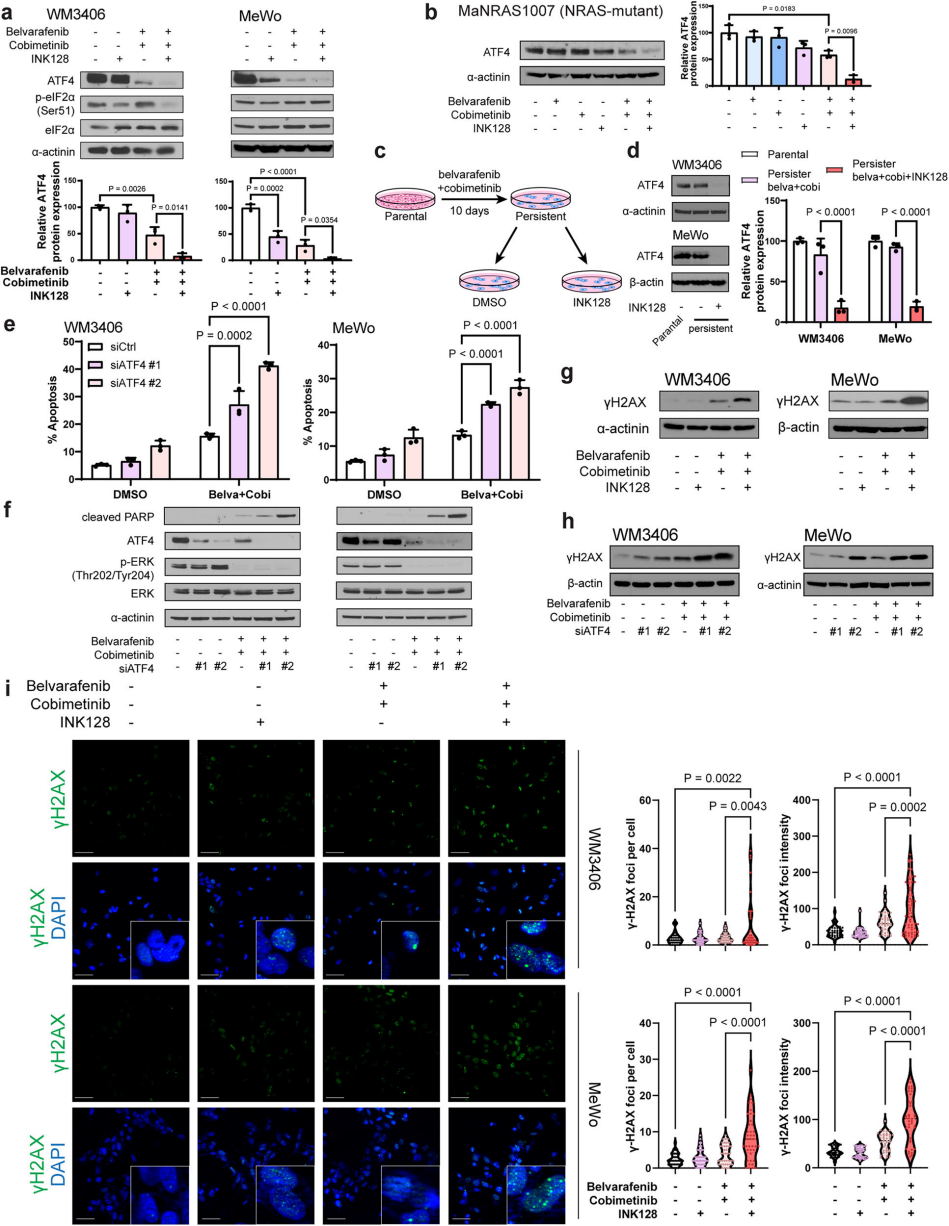
The triple therapy induces apoptosis of melanoma cells through inhibition of ATF4 and induction of DNA damage. **a** Top: Western blot analysis of the indicated proteins in WM3406 (left) and MeWo (right) cells following the treatment of INK128, belvarafenib plus cobimetinib, or the triple therapy. Bottom: Relative quantification of ATF4 protein following indicated treatment in WM3406 cells (left) and MeWo cells (right) (*n* = 3), showing that the triple therapy markedly suppresses ATF4 expression compared with MAPK inhibition alone. One-way ANOVA. **b** Left: Western blot analysis of ATF4 in mouse *NRAS*-mutant MaNRAS1007 melanoma cells following indicated treatment, demonstrating that the triple therapy suppresses ATF4 expression. Right: Relative quantification of ATF4 protein following indicated treatment in MaNRAS1007 cells (*n* = 3). One-way ANOVA. **c** Schematic of experimental design for (**d**). **d** Left: Western blot analysis of ATF4 in WM3406 (top) and MeWo (bottom) cells following belvarafenib plus cobimetinib treatment for 10 days and INK128 treatment for 2 days. Right: Relative quantification of ATF4 protein following indicated treatment in WM3406 cells and MeWo cells (*n* = 3), showing that mTOR inhibition suppresses ATF4 expression in belvarafenib plus cobimetinib persister cells. One-way ANOVA. **e** Percent apoptosis detected in DMSO- or belvarafenib plus cobimetinib-treated WM3406 (left) or MeWo (right) cells, showing that ATF4 depletion enhances apoptosis induced by MAPK inhibition. ATF4 was knocked down prior to belvarafenib plus cobimetinib treatment (*n* = 3). Two-way ANOVA. **f** Western blot analysis of the indicated proteins in WM3406 (left) and MeWo (right) cells with ATF4 knockdown or belvarafenib plus cobimetinib treatment, showing that ATF4 depletion enhances apoptosis induced by MAPK inhibition. **g** Western blot analysis of γH2AX (phosphorylated H2AX at Ser 139) in WM3406 (left) and MeWo (right) cells following indicated treatment, or **h** with ATF4 knockdown and belvarafenib plus cobimetinib treatment, showing increased DNA damage upon mTOR inhibition or the suppression of ATF4 in cells treated with belvarafenib plus cobimetinib. **i** (Left) Representative images of WM3406 (top) and MeWo (bottom) cells following the indicated treatment for 18 h and processed for γH2AX (green) immunofluorescence. Nuclei were counterstained with DAPI (scale bars: 50 μm). (Right) Quantification of γH2AX foci per cell (left) and γH2AX foci intensity (right) in WM3406 (top) and MeWo (bottom) cells following the indicated treatment (*n* = 3), confirming increased DNA damage. 40 cells per condition were randomly quantified. One-way ANOVA. All data represent mean ± SD.

Generally, ISR promotes the phosphorylation of eIF2α at serine 51, inhibiting translation initiation and leading to upregulation of ATF4 [31]. The triple therapy resulted in an inhibition of ATF4 protein expression in WM3406 and MeWo cells (Fig. 3a), without any consistent changes between cell lines in the phosphorylation status of eIF2α (Fig. 3a), which is also supported by a study showing mTORC1 controls ATF4 independently of changes in eIF2α phosphorylation [19]. Similar phenotypes were observed in MaNRAS1007, a murine *NRAS*-mutant melanoma cell line [32, 33] (Fig. 3b and Supplementary Fig. 2g).

Melanoma shows intratumor heterogeneity in the activity of MAPK signaling [34], which we hypothesize, potentially regulates ATF4 level. Thus, we next reanalyzed a spatially resolved, unsupervised transcriptomics dataset generated from tumors induced in an *NRAS*-mutant melanoma mouse model (Supplementary Fig. 3), from which the MaNRAS1007 cell line was derived [35]. Using previously defined pathway-specific gene signatures [36, 37], we noted that the tumor sample with the highest overall *ATF4* expression also had the highest MAPK- and mTOR-pathway activity (Supplementary Fig. 3a–f), consistent with prior findings that both the MAPK and mTOR pathways drive ATF4 expression in melanoma [30, 38]. Next, we applied our pathway activity analysis on the spatial transcriptomics data to assess the gene expression profile in each spatially localized environment (region of interest, ROI) (Supplementary Fig. 3a–f). As expected, the activities of MAPK and mTOR pathways were positively correlated (Supplementary Fig. 3g), supporting both pathways being simultaneously activated through common driver mutations (i.e., *NRAS*) in melanoma [39]. Interestingly, ROIs with high MAPK activity (pink and green, top 20 percentile) showed the highest *ATF4* expression, regardless of their mTOR activity. On the contrary, in MAPK^lo^ regions, high mTOR activity (top 20 percentile) is associated with retained *ATF4* expression compared with MAPK^hi^ ROIs, whereas MAPK^lo^ mTOR^lo^ regions had the lowest level of *ATF4* (Supplementary Fig. 3g). Similarly, compared with ROIs with high MAPK signaling activity, those with low MAPK signaling activity showed stronger correlation between ISR and mTOR pathway activity (Supplementary Fig. 3h). Together, these data suggest that while both the MAPK and mTOR pathways facilitate ATF4 expression [30, 38], elevated mTOR activity may compensate for MAPK suppression by maintaining *ATF4* levels and driving ISR signaling.

Given the importance of the ATF4-governed ISR in melanoma drug tolerance and resistance[20], we hypothesized that ATF4 may be essential for the survival of melanoma persister cells during combined belvarafenib plus cobimetinib therapy, which ultimately leads to resistance [40]. Although a 24-h treatment with belvarafenib plus cobimetinib repressed ATF4 expression (Fig. 3a, b), when WM3406 and MeWo cells were treated with belvarafenib plus cobimetinib for 10 days, at the timepoint when only persister cells remained (Figs. 1a and 3c), ATF4 expression was no longer impacted by belvarafenib plus cobimetinib (Fig. 3d). Importantly, INK128 was still able to efficiently downregulate ATF4 (Fig. 3d), consistent with the spatial transcriptomic data suggesting that dual blockade of MAPK and mTOR most efficiently decreases ATF4 expression (Supplementary Fig. 3h). ATF4 knockdown using short interfering RNA (siRNA) did not compromise cell survival (Fig. 3e, f), but *ATF4*-depleted cells were more sensitive to belvarafenib plus cobimetinib-induced apoptosis (Fig. 3e, f). Together, these data revealed an ATF4-dependent mechanism of tolerance in the MAPKi-induced melanoma persister cells. We suggest then that mTOR blockade enhances the efficacy of MAPKi by potentiating and sustaining ATF4 suppression.

Our transcriptomic data identified the “DNA damage response” and “double-strand break repair” pathways as being downregulated in the triple therapy-treated cells (Fig. 2a and Supplementary Fig. 2e). Given that impaired DNA double-strand break repair can lead to excessive DNA damage and increased apoptosis [41], we next examined markers of DNA damage, specifically γH2AX, a well-established read-out of DNA double-strand breaks (DSBs) [42]. Belvarafenib plus cobimetinib consistently induced γH2AX, with INK128 further enhancing this effect (Fig. 3g, i). Importantly, knockdown of *ATF4* also resulted in enhanced γH2AX formation in cells treated with combined belvarafenib plus cobimetinib (Fig. 3h), phenocopying the effect observed with the mTOR inhibitor (Fig. 3g, i). Altogether, our data suggest that mTOR inhibition potentiates belvarafenib plus cobimetinib-induced DNA damage through a mechanism downstream of ATF4.

### The triple therapy suppresses MTHFD2 downstream of ATF4

**Fig. 4:**
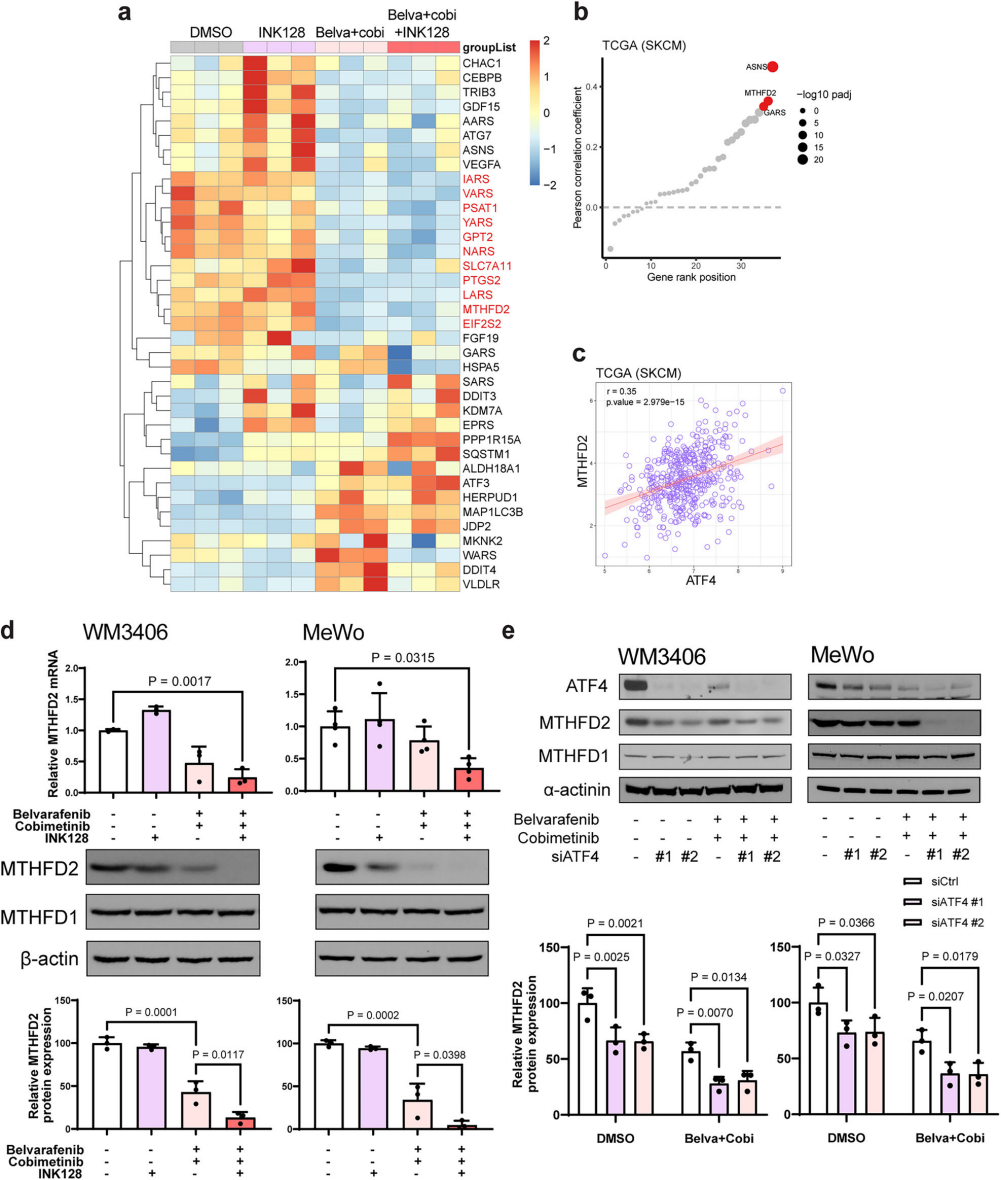
The triple therapy suppresses MTHFD2 downstream of ATF4. **a** Heatmap showing the expression of ATF4 target genes in drug-treated WM3406 cells relative to DMSO-treated WM3406 cells. **b** The plot showing the correlation between the expression of ATF4 target genes and that of *ATF4* as assessed by Pearson correlation. Red dots indicate a correlation coefficient larger than 0.3. **c** The scatter plot showing the positive correlation between the expression of *MTHFD2* and *ATF4*. Pearson correlation. **d** Top: Fold change of *MTHFD2* transcripts following indicated treatment in WM3406 cells (left) and MeWo cells (right) (*n* = 3), showing that the triple therapy significantly suppresses *MTHFD2* mRNA expression. *RPLP0* was used as a reference gene. One-way ANOVA. Middle: Western blot analysis of the indicated proteins in WM3406 (left) and MeWo (right) cells following the treatment of INK128, belvarafenib plus cobimetinib, or the triple therapy. Bottom: Relative quantification of MTHFD2 protein expression following the indicated treatment in WM3406 cells (left) and MeWo cells (right), demonstrating that triple therapy reduces MTHFD2 protein expression in melanoma cells (*n* = 3). One-way ANOVA. **e** Top: Western blot analysis of the indicated proteins in WM3406 (left) and MeWo (right) cells with ATF4 knockdown or belvarafenib plus cobimetinib treatment. Bottom: Relative quantification of MTHFD2 protein following indicated treatment in WM3406 cells (left) and MeWo cells (right) (*n* = 3), showing that ATF4 depletion suppresses MTHFD2 expression and supporting MTHFD2 as a downstream effector of ATF4 signaling. Two-way ANOVA. All data represent mean ± SD.

As an important transcription factor of ISR signaling, ATF4 target genes have been previously documented, wherein each target is evaluated and classified based on high, medium, or low confidence [31]. We interrogated our RNA sequencing data for the expression of 37 high-confidence ATF4 target genes and found that several were downregulated by the triple therapy (Fig. 4a and Supplementary Table 2). We next interrogated human melanoma data from The Cancer Genome Atlas (TCGA) for correlations between the 37 ATF4 target genes and *ATF4* expression (Fig. 4b). Among the candidate genes of interest, *methylenetetrahydrofolate dehydrogenase 2 (MTHFD2)* was noteworthy. MTHFD2 is an enzyme that functions in mitochondrial one-carbon metabolism in the tetrahydrofolate (THF) cycle, which is essential for nucleotide synthesis and helps provide building blocks for subsequent DNA repair [43], making it an attractive ATF4 downstream candidate for subsequent evaluation. *MTHFD2* was repressed by the triple therapy in our RNAseq data, and its expression correlated with ATF4 expression in the TCGA dataset (Fig. 4a, c). MTHFD2 mRNA and protein were decreased in the triple therapy-treated cells (Fig. 4d). Given our data in persister cells showing that ATF4 is only repressed by the addition of INK128 to belvarafenib plus cobimetinib-treated cells, we next assessed MTHFD2 expression under similar culture conditions. In WM3406 and MeWo persister cells, MTHFD2 expression was no longer impacted by belvarafenib plus cobimetinib (Supplementary Fig. 4), but INK128 downregulated MTHFD2 expression (Supplementary Fig. 4). Further supporting ATF4 as a regulator of MTHFD2 expression, knockdown of ATF4 led to a decrease in MTHFD2 protein, while MTHFD1, which is independent of ATF4, remained unaffected (Fig. 4e).

### Silencing of MTHFD2 in belvarafenib and cobimetinib combination-treated melanoma cells phenocopies the triple therapy by inhibiting purine biosynthesis and increasing DNA damage

**Fig. 5:**
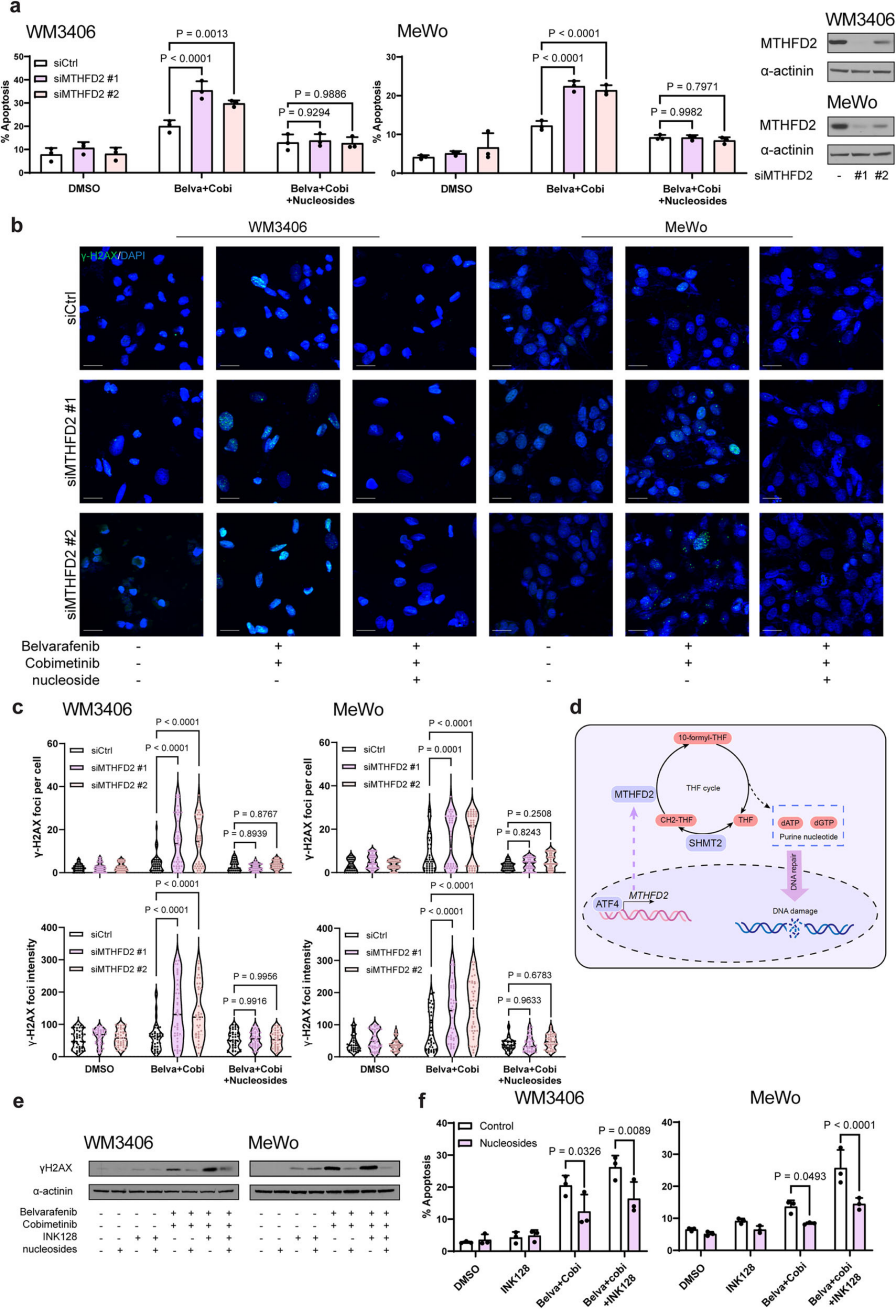
The silencing of MTHFD2 sensitizes melanoma cells to belvarafenib and cobimetinib combination therapy by inhibiting purine biosynthesis and increasing DNA damage. **a** Percent apoptosis detected in DMSO- or belvarafenib plus cobimetinib- or belvarafenib plus cobimetinib plus nucleosides-treated WM3406 (left) or MeWo (middle) cells. MTHFD2 was knocked down prior to belvarafenib plus cobimetinib treatment (*n* = 3), showing that depletion of MTHFD2 enhances apoptosis induced by MAPK inhibition and that nucleoside supplementation rescues this effect. Two-way ANOVA. Right: Western blot analysis of MTHFD2 in WM3406 (top) and MeWo (bottom) cells with MTHFD2 knockdown. **b** (Left) Representative images of WM3406 (left) and MeWo (right) cells following indicated treatment for 18 h and processed for γH2AX (green) immunofluorescence. Nuclei were counterstained with DAPI (scale bars: 25 μm). **c** Quantification of γH2AX foci per cell (top) and γH2AX foci intensity (bottom) in WM3406 (left) and MeWo (right) cells following indicated treatment (*n* = 3), demonstrating increased DNA damage upon MTHFD2 depletion and the rescue by nucleoside supplementation. 40 cells per condition were randomly quantified. Two-way ANOVA. **d** Schematic diagram showing the one-carbon metabolism and nucleotide biosynthesis in melanoma cells. **e** Western blot analysis of γH2AX and **f** percent apoptosis detected in WM3406 (left) or MeWo (right) cells following belvarafenib, cobimetinib, INK128 treatment with or without nucleosides supplementation (*n* = 3), showing that nucleoside supplementation reduces DNA damage and apoptosis induced by the triple therapy. Two-way ANOVA. All data represent mean ± SD.

Given the essential role of MTHFD2 in nucleotide synthesis and DNA repair, we sought to explore the link between the ATF4-MTHFD2 axis and the triple therapy-induced DNA damage. Similar to the data obtained in *ATF4*-silenced melanoma cells, *MTHFD2* knockdown sensitized melanoma cells to belvarafenib plus cobimetinib treatment, with a significantly increased level of DNA damage (Fig. 5a–c).

THF cycle produces one-carbon formyl groups for various cellular processes, including de novo purine synthesis. MTHFD2 in the THF cycle can oxidize CH2-THF to 10-formyl THF, which participates in multiple steps in purine synthesis [44–46]. Prior work has shown that the amount of purine nucleotides is greatly reduced in *MTHFD2* knockdown cancer cells [47] and that impairing nucleotide biosynthesis results in an insufficient supply of metabolites for DNA repair [48]. Nucleoside supplementation, which bypasses the need for MTHFD2 in nucleotide biosynthesis (Fig. 5d), protected *MTHFD2*-depleted cells from the apoptosis induced by belvarafenib plus cobimetinib (Fig. 5a), and significantly reduced γH2AX levels in these conditions (Fig. 5b, c). Similarly, the addition of nucleosides also attenuated the DNA damage observed in belvarafenib plus cobimetinib-treated cells and triple therapy-treated cells (Fig. 5e), resulting in an overall reduction of apoptosis following these treatments (Fig. 5f). Together, these data suggest that the triple therapy induces DNA damage and apoptosis in melanoma cells via blocking the ATF4-MTHFD2 axis-mediated DNA repair pathways.

### The triple therapy decreases tumor outgrowth and improves survival of *NRAS*-mutant melanoma-bearing mice in vivo

**Fig. 6:**
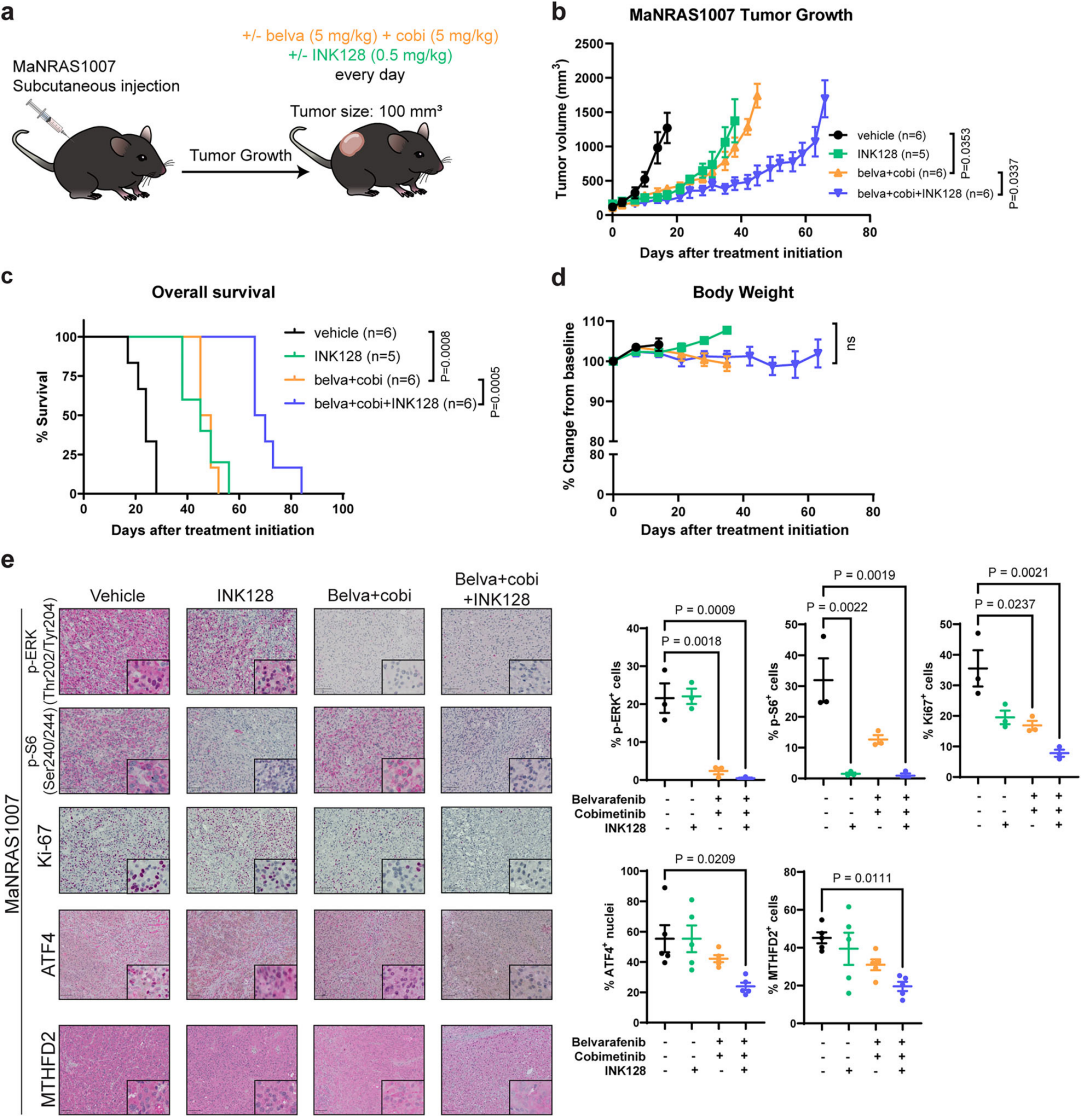
The triple therapy is effective to treat *NRAS*-mutant melanoma in vivo. **a** Schematic of experimental design (gavage of drugs) (**b**–**d**). **b** Tumor growth curve comparing MaNRAS1007 melanomas grown on C57BL/6N host mice with indicated treatments, showing that the triple therapy significantly suppresses tumor outgrowth compared with vehicle or belvarafenib plus cobimetinib. **c** Kaplan–Meier curves showing overall survival (OS) of mice bearing MaNRAS1007 melanomas with indicated treatments, demonstrating that triple therapy prolongs overall survival. Log-rank test. **d** Relative weight change (% initial body weight prior to treatment) in mice receiving indicated treatments, indicating that the triple therapy is well tolerated. **e** Representative images (left) and percentages (right) of p-ERK, p-S6, Ki67, ATF4, and MTHFD2-positive melanoma cells in primary melanoma sections with indicated treatment. The positive signals (pink) were indicated by magenta red, and tissues were counterstained with Hematoxylin (purple). Three melanomas from each treatment arm were stained for p-ERK, p-S6, and Ki67, to confirm the on-target effect of the drugs (day 14; *n* = 3; scale bars: 100 μm). The percentages of ATF4-positive cell nuclei and MTHFD2-positive cells were quantified (day 20; *n* = 5; scale bars: 100 μm), demonstrating suppression of the ATF4-MTHFD2 axis in melanomas treated with the triple therapy. All data represent mean ± SEM.

To evaluate the triple therapy in vivo, we used the MaNRAS1007 murine melanoma model [32]. Tumor-bearing mice were randomized into 4 treatment arms: vehicle, INK128 (0.5 mg/kg/daily), belvarafenib (5 mg/kg/daily) plus cobimetinib (5 mg/kg/daily), and the triple therapy (Fig. 6a). We tailored the doses of each drug to ensure on-target engagement and optimize therapeutic efficacy, while minimizing potential toxicity. Melanoma outgrowth was substantially inhibited in the INK128 monotherapy, and as expected, the tumors responded to belvarafenib plus cobimetinib [11] (Fig. 6b). However, the tumors gradually progressed at approximately day 40 of the belvarafenib plus cobimetinib treatment, suggesting the emergence of drug resistance (Fig. 6b). Notably, tumor control was significantly extended in the mice administered the triple therapy, compared with those that received INK128 monotherapy or the belvarafenib plus cobimetinib dual therapy. Importantly, the triple therapy cohort showed significantly improved overall survival (OS), compared with any other treatment groups (Fig. 6c). No significant weight loss was observed, indicating good tolerability of the therapy (Fig. 6d). These data suggest that mTOR inhibition may eliminate a substantial proportion of melanoma cells that resist combined belvarafenib plus cobimetinib (Fig. 6b and Supplementary Fig. S4).

Belvarafenib plus cobimetinib inhibited p-ERK levels, and INK128 repressed p-S6 expression in the tumors from treated mice (Fig. 6e). There were fewer Ki67-positive cells in the triple therapy-treated melanomas (Fig. 6e), suggesting an inhibition of melanoma proliferation. Finally, we found that nuclear ATF4 expression and the expression of MTHFD2 were lower in the triple therapy cohort, suggesting impaired ATF4-MTHFD2 signaling in vivo (Fig. 6e).

### mTOR inhibition can overcome the resistance to the combination therapy of belvarafenib and cobimetinib in *NRAS*-mutant melanoma

**Fig. 7:**
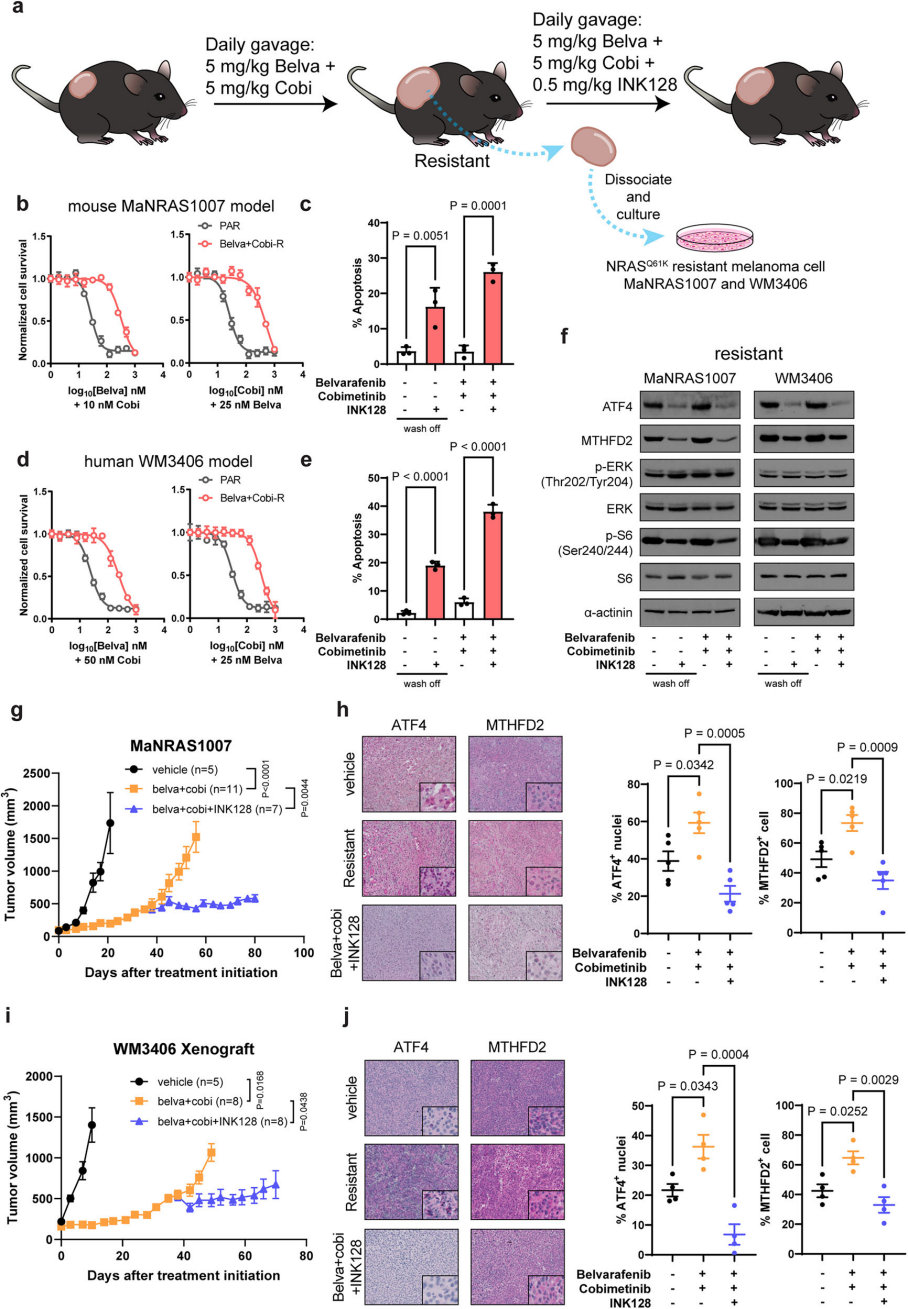
Targeting mTOR activity overcomes the resistance of belvarafenib and cobimetinib combination therapy in *NRAS*-mutant melanoma. **a** Schematic of experimental design for (**b**–**i**). **b** Left: IC_50_ curve for belvarafenib inhibition of MaNRAS1007 tumor dissociated parental (IC_50_ = 27.20 nM) and drug-resistant (IC_50_ = 320.1 nM) tumor-derived cells with the treatment of 10 nM of cobimetinib (*n* = 4). Right: IC_50_ curve for cobimetinib inhibition of MaNRAS1007 tumor dissociated parental (IC_50_ = 26.19 nM) and drug-resistant (IC_50_ = 504.4 nM) cells with the treatment of 25 nM of belvarafenib (*n* = 4). **c** Percent apoptosis in belvarafenib plus cobimetinib-resistant MaNRAS1007 cells following INK128 treatment (*n* = 3), showing MaNRAS1007 tumor-derived belvarafenib plus cobimetinib-resistant cells undergo apoptosis in response to mTOR inhibition. Two-sided unpaired *t*-test. **d** Left: IC_50_ curve for belvarafenib inhibition of WM3406 tumor dissociated parental (IC_50_ = 23.88 nM) and drug-resistant (IC_50_ = 239.5) cells with the treatment of 50 nM of cobimetinib (*n* = 4). Right: IC_50_ curve for cobimetinib inhibition of WM3406 tumor dissociated parental (IC_50_ = 29.69 nM) and drug-resistant (IC_50_ = 316.7 nM) cells with the treatment of 25 nM of belvarafenib (*n* = 4). **e** Percent apoptosis in belvarafenib plus cobimetinib-resistant WM3406 tumor-derived cells following INK128 treatment (*n* = 3), showing that MAPK inhibitor resistant cells undergo apoptosis in response to mTOR inhibition. Two-sided unpaired *t*-test. **f** Western blot analysis of the indicated protein in MaNRAS1007 (left) or WM3406 (right) resistant cells following INK128 treatment with or without washing off belvarafenib plus cobimetinib, showing suppression of the ATF4 and MTHFD2 upon mTOR inhibition. **g** Tumor growth curve comparing MaNRAS1007 melanomas grown on C57BL/6N host mice with or without belvarafenib plus cobimetinib treatments. After melanomas got resistant to belvarafenib plus cobimetinib treatments, mice were randomly split to be treated with belvarafenib plus cobimetinib treatments (orange) or the triple therapy (blue), showing that the addition of INK128 suppresses the growth of resistant tumors. Two-way ANOVA. **h** Representative images (left) and percentages (right) of ATF4 and MTHFD2-positive melanoma cells in primary melanoma sections with indicated treatment (*n* = 5; scale bars: 100 μm), demonstrating suppression of the ATF4-MTHFD2 axis in resistant tumors treated with the triple therapy. One-way ANOVA. **i** Tumor growth curve comparing WM3406 melanomas grown on NOD/SCID host mice with or without belvarafenib plus cobimetinib treatments. After melanomas became resistant to belvarafenib plus cobimetinib treatments, mice were randomly split to treat with belvarafenib plus cobimetinib treatments (orange) or the triple therapy (blue), showing that triple therapy suppresses tumor growth after resistance to belvarafenib plus cobimetinib has developed. Two-way ANOVA. **j** Representative images (left) and percentages (right) of ATF4 and MTHFD2-positive melanoma cells in primary melanoma sections with indicated treatment (*n* = 4; scale bars: 100 μm), confirming inhibition of the ATF4-MTHFD2 axis in resistant tumors treated with the triple therapy. One-way ANOVA. In all the IHC data, the positive signals (pink) were indicated by magenta red, and tissues were counterstained with Hematoxylin (purple). **b**–**e** represent mean ± SD. **g**–**j** represent mean ± SEM.

We next tested whether blocking mTOR would be of clinical benefit once therapeutic resistance was acquired using cell lines derived from pre-clinical mouse models (Fig. 7a). For this, mice bearing MaNRAS1007 or WM3406 melanomas were treated with belvarafenib plus cobimetinib; after 35 days, the tumors began to progress, indicating acquired resistance. To confirm resistance to belvarafenib plus cobimetinib, we dissociated the parental and dual drug-resistant melanomas (i.e., derived from MaNRAS1007 or WM3406 melanomas) and tested them in vitro. As expected, the resistant cell lines did not respond to belvarafenib and cobimetinib (Fig. 7b, d). However, treatment with INK128 significantly induced apoptosis in these resistant cell lines (Fig. 7c, e), along with a repression in ATF4 and MTHFD2 protein expression (Fig. 7f), supporting its potential as a salvage therapy. Therefore, we next treated these belvarafenib plus cobimetinib-resistant MaNRAS1007 and WM3406 melanomas with INK128, while keeping the mice on belvarafenib plus cobimetinib, and observed that INK128 inhibited tumor outgrowth of both melanoma models (Fig. 7g, i). Consistently, melanomas that were resistant to belvarafenib plus cobimetinib expressed higher ATF4 and MTHFD2. INK128 reduced the levels of ATF4 and MTHFD2 in these belvarafenib plus cobimetinib-resistant tumors (Fig. 7h, j). Taken together, our results provide a rationale to inhibit mTOR activity to overcome acquired resistance to combined belvarafenib plus cobimetinib.

## Discussion

In this present study, we showed that mTOR inhibition can augment therapeutic responses to combined belvarafenib and cobimetinib in cell culture models and pre-clinical mouse models of hard-to-treat and therapy-resistant melanoma, without associated toxicity in non-malignant cell models or mice. While our study supports the established crosstalk between the MAPK and mTOR pathways in regulating ATF4 activity [14, 19, 49, 50], it also uncovers a novel therapeutic mechanism: combining pan-RAF inhibitors with MEK and mTOR inhibitors effectively suppresses the ATF4-MTHFD2 axis, disrupting purine synthesis and impairing DNA repair. We provide evidence that this multi-targeted approach offers a powerful strategy to overcome resistance and enhance anti-tumor efficacy in MAPK-driven cancers.

Our findings are aligned with prior work demonstrating that combined belvarafenib and cobimetinib therapy exhibits clinical activity in patients with *NRAS*-mutant melanomas (NCT03284502), with an overall response rate of 38.5% [11, 12]. These results highlight the therapeutic potential of MAPK inhibition in this molecular subtype of melanoma, which historically has limited targeted therapy options. INK128 has also reached clinical testing and is well tolerated (NCT02412722, NCT02197572), thus providing an opportunity to assess whether mTOR inhibition improves the efficacy of combined belvarafenib and cobimetinib. Notably, in *NRAS*-mutant melanomas treated with belvarafenib plus cobimetinib, acquired resistance can develop. We have shown that drug-resistant melanomas maintain ATF4 signaling and can be resensitized by pharmacologic inhibition of mTOR (Fig. 7). Therefore, INK128 may be tested first in patients who have progressed on combination belvarafenib plus cobimetinib therapy.

Oncogenic MAPK signaling induces the expression of ATF4, which plays a central role in stress response and cell survival [30]. In *NRAS* and *NF1*-mutant melanomas, we demonstrate that ATF4 expression is partially repressed following the blockade of MAPK signaling by belvarafenib plus cobimetinib (Fig. 3a, b), adding an important perspective to an earlier study [38]. Moreover, we showed that this suppression is incomplete, suggesting that additional signaling pathways may sustain ATF4 expression under MAPK-targeted therapy. Consistent with this notion, we found that pharmacologic inhibition of mTOR in the context of belvarafenib plus cobimetinib further suppressed ATF4 (Fig. 3a, b). This finding is in agreement with prior work showing that the mTOR pathway is required for efficient MAPK-mediated ATF4 induction [30].

Drug-persistent melanoma cells in vitro and resistant melanomas in vivo maintain ATF4 expression, which can be impaired upon inhibiting mTOR to overcome therapeutic resistance (Figs. 3d and 7c, f). These results indicate the involvement of the ATF4 pathway in belvarafenib plus cobimetinib persister cells, consistent with a pro-survival role. Indeed, activation of the ATF4 pathway represents an evolutionarily conserved general stress response that may function in adaptation to MAPK inhibitors [20]. Such stress-induced rewiring of signaling networks may create compensatory survival pathways that support drug resistance, and that is not a melanoma-specific therapeutic challenge. This highlights the importance of identifying cooperative and synergistic drug combinations, perhaps using computational methods, to disrupt such adaptive signaling networks [51].

Given the importance of ATF4 in drug persistence and resistance, the widespread role of ATF4 target genes in mitochondrial function, autophagy, pro-apoptotic pathway, and tRNA biosynthesis should not be neglected [31]. Herein, our data in melanoma models support that MTHFD2 is a target of ATF4, consistent with strong evidence from human and murine studies indicating that ATF4 regulates MTHFD2 transcription [52–55]. Moreover, activated mTOR leads to upregulated MTHFD2 and higher activity of purine synthesis, while knockdown of ATF4 decreases MTHFD2, which further indicates that, as a metabolic effector of mTOR [49], ATF4 is required for the induction of MTHFD2-mediated purine synthesis [56]. Although previous studies have shown that targeting mTOR or ATF4 downregulates MTHFD2 [19, 49], our study provides the first evidence that MTHFD2 regulates purine synthesis and DNA damage repair, thereby modulating the response to MAPK-targeted therapy in non-*BRAF*-mutant melanoma.

Our finding of the upregulation of ATF4 and MTHFD2 in melanomas that are resistant to combined belvarafenib and cobimetinib therapy raises the question of how ATF4 and MTHFD2 impact drug resistance. MTHFD2-mediated purine synthesis produces free nucleotides as building blocks for subsequent DNA synthesis and repair [57, 58]. Increased nucleotide availability may therefore facilitate tumor cell survival under therapeutic stress. Consistent with this possibility, a previous study showed that overexpressing MTHFD2 results in MAPK signaling activation in bladder cancer [59]. Reactivated MAPK signaling is a hallmark of targeted therapy resistance in melanoma cells, indicating a potential link between MTHFD2 and drug resistance. In support of these speculations, MTHFD2 is found to be critical for lung adenocarcinoma that is resistant to the EGFR inhibitor, likely through increasing purine nucleotide [47]. MTHFD2 knockdown reduces folate-mediated one-carbon metabolism and purine synthesis in lung cancer cells, increasing the sensitivity to EGFR inhibitor gefitinib [47]. In addition, it is widely accepted that impairing purine synthesis causes DNA repair deficiency, which contributes to the efficacy of radiation therapy in glioblastoma [48]. In line with these concepts, we show that inhibiting mTOR or knocking down *MTHFD2* increases DNA damage in combined belvarafenib and cobimetinib therapy (Fig. 3i), and nucleoside supplementation decreases the accumulation of DNA damage (Fig. 5). These results are consistent with recent work identifying that the supplementation of nucleoside promotes DNA repair in glioblastoma, where inhibiting GTP synthesis impairs DNA repair and sensitizes resistant glioblastoma cells to radiotherapy [48, 60].

Some limitations of this study include the lack of access to melanoma samples from patients who have received pan-RAF plus MEK combination therapy. We were thus unable to probe for the expression of ATF4 or MTHFD2 and correlate it with the expression of activated mTOR. We also did not determine whether mTOR inhibitors would augment the anti-tumor benefit of other pan-RAF inhibitor combination therapies. Finally, future work is still needed to understand whether the immune microenvironment differs in mice treated with belvarafenib plus cobimetinib versus the triple therapy.

In summary, we provide evidence that pharmacologic inhibition of mTOR shows efficacy in pre-clinical melanoma models when combined with belvarafenib and cobimetinib, via a mechanism involving the blockade of ATF4-MTHFD2 axis-regulated purine synthesis, which results in DNA damage and apoptosis. Our findings support the concept that rationally designed small-molecule combinations targeting complementary signaling pathways may improve therapeutic efficacy and overcome adaptive therapeutic resistance in cancer [61].

## Supplementary information

Supplementary file

Figure S1

Figure S2

Figure S3

Figure S4

Figure S5

aj_checklist

Original Western blots

## Data Availability

The RNA sequencing data used in this study are available in the GSE301844. All data reported in this article will be shared by the lead contact upon request. Any additional information required to reanalyze the data reported in this article is available from the lead contact upon request.
