# Supplementary material for: mTOR inhibition enhances the antitumor efficacy of pan-RAF-MEK blockade by inhibiting the ATF4-MTHFD2 pathway
Source: Cell Death Dis. 2026 May 6;17(1):600. doi: 10.1038/s41419-026-08836-5 (PMC13315934; doi:10.1038/s41419-026-08836-5)
Supplement: Supplementary file 1 — Supplementary file [file 41419_2026_8836_MOESM1_ESM.docx]

**Supplementary Fig. 1 Blocking the mTOR pathway with INK128 augments therapeutic responses to belvarafenib plus cobimetinib therapy in melanoma cells.**

**a,** Left: IC_50_ curve for belvarafenib inhibition of WM3406 cell (IC_50_ = 35.68 nM), BLM cell (IC_50_ = 59.30 nM), MeWo cell (IC_50_ = 73.83 nM) and HBL cell (IC_50_ = 142.4 nM) with the treatment of 50 nM of cobimetinib (n=4). Right: IC_50_ curve for cobi inhibition of WM3406 cell (IC_50_ = 60.19 nM), BLM cell (IC_50_ = 71.13 nM), MeWo cell (IC_50_ = 88.81 nM) and HBL cell (IC_50_ = 137.0 nM) with the treatment of 25 nM of belva (n=4). **b,** Colony number of YUGOE cells following belvarafenib (25 nM), cobimetinib (50 nM) and INK128 (25 nM) treatment, showing that the addition of INK128 enhances the growth-suppressive effects of belvarafenib plus cobimetinib (top) and western blot analysis to confirm the on-target effect of indicated drugs (bottom) (n=3). One-way ANOVA. **c,** Percent apoptosis measured as the sum of PI^+^/Annexin V^+^ and PI^-^/Annexin V^+^ populations (top) and western blot analysis to confirm the induction of apoptosis (cleaved PARP) in melanoma cells (WM3623) and immortalized melanocytes (MelST and Melan-A) (bottom) (n=3), demonstrating that triple therapy induces apoptosis of melanoma cells but not melanocytes. One-way ANOVA. Data are presented as mean ± SD. Number of biological replicates is indicated in each graph.

**Supplementary Fig. 2 RNA sequencing data and pathway analysis from WM3406-treated cells.**

**a,** Schematic of the treatment on NRAS-mutant WM3406 melanoma cell line for RNA sequencing. **b,** Heatmap showing genes that were altered in drug treated cells relative to DMSO treated cells. GSEA for DEGs of triple therapy treated cells in **c,** cell cycle, **d,** DNA replication and **e,** double-strand break repair. **f,** GSEA for DEGs of belvarafenib plus cobimetinib treated cells in cellular response to stress. **g,** Apoptosis (left graph) and western blot analysis (shown right) done in MaNRAS1007 cells with indicated treatment (n=3), demonstrating increased apoptosis upon triple therapy compared with belvarafenib plus cobimetinib. Data are presented as mean ± SD.

**Supplementary Fig. 3 Spatially mapping ATF4 expression, MAPK and mTOR pathway activity in *NRAS*-mutant melanoma.**

Spatially resolved quantification of **a** *Atf4* expression, **c** MPAS score and **e** mTOR pathway activity score by label transfer of scRNA-seq data onto Visium spots (sample 1,2,3). **b**, **d** and **f** are quantification of **a**, **c** and **e**, respectively. Boxes extend from the 25^th^ to 75^th^ percentile. The middle line represents the median. Whiskers represent 10^th^ to 90^th^ percentile. One-way ANOVA. **g**, (Left) The scatter plot showing the correlation between the MPAS and mTOR pathway activity score, highlighting coordinated activation of MAPK and mTOR signaling. The 20^th^ percentile cutoff line set to distinguish cells with different MAPK and mTOR activity. Pearson correlation. (right) The violin plot showing the expression of *Atf4* in cell populations with distinct MAPK and mTOR pathway activity, demonstrating higher ATF4 expression in cells with elevated MAPK and mTOR signaling. **h**, The scatter plot showing the correlation between the mTOR pathway activity score and ISR score in MAPK^hi^ (top) and MAPK^lo^ (bottom) cell population. Pearson correlation.

**Supplementary Fig. 4 mTOR inhibitor suppresses MTHFD2 in belvarafenib plus cobimetinib-persistent melanoma cells.**

Western blot analysis of the indicated proteins in WM3406 (left) and MeWo (right) cells following belvarafenib plus cobimetinib treatment for 10 days and INK128 treatment for 2 days.

**Supplementary Fig. 5 The triple therapy demonstrates anti-tumoral activity in MaNRAS1007 melanoma models.**

Individual growth of MaNRAS1007-derived melanomas.

**Supplementary Table 1.** Detailed list of genes in highlighted GO terms, related to Fig. 2b.

| **DNA damage response** | **double-strand break repair** | **cellular response to stress** |
| --- | --- | --- |
| *WRAP53* | *WRAP53* | *LIG1* |
| *ERCC1* | *ERCC1* | *TNFRSF19* |
| *DPF1* | *DPF1* | *NDP* |
| *CDC7* | *CDC7* | *WNK3* |
| *CXCL12* | *GEN1* | *FANCM* |
| *GEN1* | *DCLRE1B* | *ZNF365* |
| *POLE3* | *TWIST1* | *MEIOB* |
| *DCLRE1B* | *RPA3* | *SDF2L1* |
| *EEF1E1* | *FANCM* | *PDGFRA* |
| *MASTL* | *ZNF365* | *POLD1* |
| *TWIST1* | *MEIOB* | *PPARGC1B* |
| *RPA3* | *POLA1* | *MYC* |
| *LIG1* | *RUVBL1* | *POLA1* |
| *FANCM* | *RFWD3* | *RUVBL1* |
| *ZNF365* | *MSH2* | *FAM111A* |
| *MEIOB* | *EYA1* | *POLE* |
| *POLD1* | *FANCB* | *RFWD3* |
| *MYC* | *MMS22L* | *OPRD1* |
| *POLA1* | *EME1* | *SUV39H2* |
| *RUVBL1* | *RMI1* | *MFSD2A* |
| *FAM111A* | *FIGNL1* | *PIF1* |
| *POLE* | *PARPBP* | *CDK2* |
| *RFWD3* | *TIMELESS* | *RBL1* |
| *PIF1* | *MCM2* | *WDR4* |
| *CDK2* | *CHEK1* | *NLRP3* |
| *WDR4* | *MCM5* | *RRM1* |
| *RRM1* | *MCM6* | *EZH2* |
| *BARD1* | *CHEK2* | *BARD1* |
| *MSH2* | *TONSL* | *TERT* |
| *E2F7* | *BRCA2* | *MSH2* |
| *EYA1* | *AUNIP* | *E2F7* |
| *IER3* | *MCM8* | *EYA1* |
| *UNG* | *HMGA2* | *IER3* |
| *POLD3* | *RAD54B* | *UNG* |
| *FANCB* | *DNA2* | *MGST1* |
| *TOP2A* | *TRIP13* | *HMGA1* |
| *RFC2* | *FOXM1* | *POLD3* |
| *RFC4* | *BRCA1* | *FANCB* |
| *TRAIP* | *RECQL4* | *MAP3K5* |
| *MMS22L* | *ANKLE1* | *CSPG5* |
| *EME1* | *MCM3* | *RNF152* |
| *TIPIN* | *BLM* | *TOP2A* |
| *FANCG* | *XRCC3* | *RFC2* |
| *RNASEH2A* | *MCM7* | *RFC4* |
| *RMI1* | *RMI2* | *TRAIP* |
| *FIGNL1* | *MCM4* | *MMS22L* |
| *PARPBP* | *RAD51* | *EME1* |
| *RFC5* | *CDCA5* | *TIPIN* |
| *TIMELESS* | *FEN1* | *DCTPP1* |
| *SUV39H1* | *FANCD2* | *FANCG* |
| *CHAF1B* | *RAD51AP1* | *RNASEH2A* |
| *MCM2* | *GINS4* | *RMI1* |
| *PTTG1* | *POLQ* | *FIGNL1* |
| *PLK1* | *XRCC2* | *PARPBP* |
| *KIF22* | *GINS2* | *RFC5* |
| *CHEK1* | *BRIP1* | *TIMELESS* |
| *MCM5* | *UHRF1* | *SUV39H1* |
| *FBXO5* | *TEX15* | *CHAF1B* |
| *MCM6* | *RAD54L* | *MCM2* |
| *CCND1* | *ESCO2* | *PTTG1* |
| *CHEK2* | *CDC45* | *TFPI2* |
| *TONSL* |  | *PLK1* |
| *BRCA2* |  | *KIF22* |
| *AUNIP* |  | *CHEK1* |
| *VRK1* |  | *MCM5* |
| *MCM8* |  | *ECT2* |
| *HMGA2* |  | *FBXO5* |
| *RAD54B* |  | *MCM6* |
| *GTSE1* |  | *CCND1* |
| *WDHD1* |  | *STOX1* |
| *DNA2* |  | *CHEK2* |
| *TRIP13* |  | *TONSL* |
| *FOXM1* |  | *BRCA2* |
| *RFC3* |  | *AUNIP* |
| *BRCA1* |  | *VRK1* |
| *RECQL4* |  | *NET1* |
| *ATAD5* |  | *MCM8* |
| *ANKLE1* |  | *RASGRF2* |
| *MCM3* |  | *HMGA2* |
| *FANCI* |  | *DHFR* |
| *BLM* |  | *FOSL1* |
| *XRCC3* |  | *RAD54B* |
| *MCM7* |  | *TNC* |
| *RMI2* |  | *SCARA5* |
| *MCM4* |  | *DHRS2* |
| *RAD51* |  | *MMP3* |
| *CDCA5* |  | *SFRP1* |
| *FEN1* |  | *GTSE1* |
| *TICRR* |  | *WDHD1* |
| *WDR76* |  | *XDH* |
| *CHAF1A* |  | *DNA2* |
| *FANCA* |  | *MYB* |
| *POLE2* |  | *TRIP13* |
| *FANCD2* |  | *FOXM1* |
| *NEIL3* |  | *RFC3* |
| *UBE2T* |  | *CCNA2* |
| *CDK1* |  | *FOXA3* |
| *PMAIP1* |  | *BRCA1* |
| *RAD51AP1* |  | *PTGS2* |
| *GINS4* |  | *F2RL1* |
| *POLQ* |  | *RECQL4* |
| *XRCC2* |  | *ATAD5* |
| *GINS2* |  | *TFEC* |
| *BRIP1* |  | *ANKLE1* |
| *UHRF1* |  | *MCM3* |
| *CLSPN* |  | *FANCI* |
| *EXO1* |  | *BLM* |
| *TEX15* |  | *XRCC3* |
| *E2F1* |  | *MCM7* |
| *RAD54L* |  | *RMI2* |
| *VAV3* |  | *MCM4* |
| *ESCO2* |  | *RAD51* |
| *CDC45* |  | *CDCA5* |
| *DTL* |  | *FEN1* |
| *MCM10* |  | *TICRR* |
|  |  | *WDR76* |
|  |  | *CHAF1A* |
|  |  | *FANCA* |
|  |  | *POLE2* |
|  |  | *FANCD2* |
|  |  | *NEIL3* |
|  |  | *UBE2T* |
|  |  | *BATF3* |
|  |  | *CDK1* |
|  |  | *PMAIP1* |
|  |  | *RAD51AP1* |
|  |  | *GINS4* |
|  |  | *POLQ* |
|  |  | *XRCC2* |
|  |  | *GINS2* |
|  |  | *BRIP1* |
|  |  | *PBK* |
|  |  | *GAP43* |
|  |  | *UHRF1* |
|  |  | *CLSPN* |
|  |  | *ETV5* |
|  |  | *EXO1* |
|  |  | *TEX15* |
|  |  | *E2F1* |
|  |  | *RAD54L* |
|  |  | *VAV3* |
|  |  | *ESCO2* |
|  |  | *CDC45* |
|  |  | *DTL* |
|  |  | *ADCY8* |
|  |  | *STC1* |
|  |  | *MCM10* |
|  |  | *EGR1* |

**Supplementary Table 2.** Detailed information of the differential expression of ATF4 target genes in triple therapy treated WM3406 cells relative to DMSO treated cells, related to Fig. 4a.

|  | **log2FoldChange** | **padj** |
| --- | --- | --- |
| *PTGS2* | -2.97221 | 1.12E-28 |
| *PSAT1* | -2.31722 | 5.07E-14 |
| *FGF19* | -1.56699 | 0.679829 |
| *MTHFD2* | -1.31614 | 4.73E-09 |
| *GPT2* | -1.13739 | 2.16E-10 |
| *GDF15* | -1.13406 | 0.000619 |
| *IARS* | -1.05885 | 8.87E-19 |
| *VARS* | -0.9462 | 7.95E-15 |
| *YARS* | -0.87706 | 1.50E-18 |
| *HSPA5* | -0.79365 | 4.59E-13 |
| *LARS* | -0.71443 | 1.34E-09 |
| *NARS* | -0.6965 | 4.42E-13 |
| *EIF2S2* | -0.64437 | 2.28E-10 |
| *SLC7A11* | -0.57595 | 0.15451 |
| *VEGFA* | -0.57257 | 0.092853 |
| *WARS* | -0.48911 | 6.35E-06 |
| *TRIB3* | -0.45597 | 0.163654 |
| *MKNK2* | -0.37519 | 0.100939 |
| *GARS* | -0.35951 | 0.025014 |
| *ASNS* | -0.35587 | 0.294096 |
| *ATG7* | -0.33503 | 0.028952 |
| *CHAC1* | -0.22413 | 0.740315 |
| *AARS* | -0.18934 | 0.239977 |
| *CEBPB* | -0.13112 | 0.71613 |
| *ALDH18A1* | -0.07349 | 0.748133 |
| *SARS* | 0.116661 | 0.498907 |
| *EPRS* | 0.338899 | 0.012657 |
| *HERPUD1* | 0.504504 | 0.001681 |
| *MAP1LC3B* | 0.529188 | 3.87E-10 |
| *DDIT3* | 0.617199 | 0.055697 |
| *VLDLR* | 0.727958 | 0.137348 |
| *KDM7A* | 1.027052 | 0.000397 |
| *SQSTM1* | 1.077674 | 3.15E-20 |
| *DDIT4* | 1.260573 | 5.56E-06 |
| *PPP1R15A* | 1.361313 | 6.86E-45 |
| *JDP2* | 1.831611 | 4.69E-11 |
| *ATF3* | 3.17252 | 1.55E-30 |

**Supplementary Table 3.** Detailed information of human cell lines, source, culture conditions, treatment, and experimental timeline.

| Cell line & source | Cultured in | Treatment | Timeline |
| --- | --- | --- | --- |
| BLM  Gifted by Dr. Ian R Watson (MUHC Research Institute) | DMEM | Belva (25 nM)  Cobi (50 nM)  INK128 (25 nM) | Apoptosis assay: 48h  Western blot (WB) for p-ERK: 2h  WB for p-S6: 2h  WB for cleaved PARP: 48h  WB for ATF4: 24h  WB for p-eIF2α: 24h  WB for MTHFD2: 48h  WB for γH2AX: 48h  qPCR for MTHFD2: 24h |
| WM3406  Gifted by Dr. April Rose (Lady Davis Institute) | RPMI  1x GlutaMax |  |  |
| MeWo  Gifted by Dr. Ian R Watson (MUHC Research Institute) | DMEM |  |  |
| HBL  Gifted by Dr. Ghanem Ghanem (Institut Jules Bordet) | Ham’s F10 |  |  |
| YUGOE  Gifted by Dr. April Rose (Lady Davis Institute) | DMEM/F12 |  |  |
| WM3623  Gifted by Dr. April Rose (Lady Davis Institute) | RPMI  1x GlutaMax |  |  |
| MelST  Gifted by Dr. Robert Weinberg (Whitehead Institute) | DMEM |  |  |
| Melan-A  Gifted by Dr. April Rose (Lady Davis Institute) | RPMI  1x GlutaMax |  |  |
| MaNRAS1007  Gifted by Dr. Lionel Larue (INSERM)  (First described in PMID: 31251472) | Ham’s F12 | Belva (25 nM)  Cobi (10 nM)  INK128 (25 nM) | Apoptosis assay: 24h  WB for p-ERK: 24h  WB for p-S6: 24h  WB for cleaved PARP: 24h  WB for ATF4: 24h |

* All cells were cultured in the indicated medium supplemented with 10% FBS and 1x Pen/Strep.

**Supplementary Table 4.** Detailed information of the primary antibodies used for western blotting and immunohistochemistry staining.

| **Target** | **Antibody full name** | **Source & Catalog #** | **Experiment** |
| --- | --- | --- | --- |
| p-ERK | Phospho-p44/42 MAPK (Erk1/2) (Thr202/Tyr204) (D13.14.4E) XP® Rabbit mAb | Cell Signaling Technology #4370 | WB, IHC |
| ERK2 | ERK 2 Antibody (C-14) | Santa Cruz, sc-154 | WB |
| p-S6 | Phospho-S6 Ribosomal Protein (Ser240/244) (D68F8) XP® Rabbit mAb | Cell Signaling Technology #5364 | WB, IHC |
| S6 | S6 Ribosomal Protein (54D2) Mouse mAb | Cell Signaling Technology #2317 | WB |
| cl-PARP | Cleaved PARP (Asp214) (D64E10) XP® Rabbit mAb | Cell Signaling Technology #5625 | WB |
| cl-PARP | Cleaved PARP (Asp214) Antibody | Cell Signaling Technology #9544 | WB |
| ATF4 | ATF-4 (D4B8) Rabbit mAb | Cell Signaling Technology #11815 | WB, IHC |
| ATF4 | Anti-ATF-4 antibody | Abcam ab216839 | WB, IHC |
| p-eIF2α | Phospho-eIF2α (Ser51) (D9G8) XP® Rabbit mAb | Cell Signaling Technology #3398 | WB |
| eIF2α | eIF2α (L57A5) Mouse mAb | Cell Signaling Technology #2103 | WB |
| MTHFD1 | MTHFD1 Polyclonal antibody | Proteintech 10794-1-AP | WB |
| MTHFD2 | MTHFD2 Polyclonal antibody | Proteintech 12270-1-AP | WB, IHC |
| γH2AX | Phospho-Histone H2A.X (Ser139) Antibody | Cell Signaling Technology #2577 | WB |
| Ki67 | Ki-67 (8D5) Mouse mAb | Cell Signaling Technology #9449 | IHC |
| α-Actinin | Anti-α-actinin Antibody (H-2) | Santa Cruz, sc-17829 | WB |
| β-Actin | Monoclonal Anti-β-Actin antibody (clone AC-15) | Sigma-Aldrich #A5441 | WB |
| GAPDH | GAPDH (D16H11) XP® Rabbit mAb | Cell Signaling Technology #5174 | WB |

**Supplementary Table 5.** siRNAs.

| **Gene name** | **siRNA** | **Duplex Sequence (5’-3’)** |
| --- | --- | --- |
| ***ATF4 (human)*** | *siATF4-1* | rGrGrUrCrArGrUrCrCrCrUrCrCrArArCrArArCrArGrCrAAG |
|  |  | rCrUrUrGrCrUrGrUrUrGrUrUrGrGrArGrGrGrArCrUrGrArCrCrArA |
|  | *siATF4-2* | rArUrArGrUrCrArGrGrArGrCrGrUrCrArArUrGrUrGrCrUTG |
|  |  | rCrArArGrCrArCrArUrUrGrArCrGrCrUrCrCrUrGrArCrUrArUrCrC |
| ***MTHFD2 (human)*** | *siMTHFD2-1* | rGrUrArUrUrCrCrArArArUrCrUrGrArUrCrArCrArGrCrAGA |
|  |  | rUrCrUrGrCrUrGrUrGrArUrCrArGrArUrUrUrGrGrArArUrArCrCrU |
|  | *siMTHFD2-2* | rGrCrUrUrGrGrGrUrArArGrUrArCrGrCrArArCrUrUrArCTT |
|  |  | rArArGrUrArArGrUrUrGrCrGrUrArCrUrUrArCrCrCrArArGrCrUrG |
| ***Negative control*** | *siCtrl* | N/A (Proprietary, AllStar Neg. Control siRNA, QIAGEN #1027281) |

**Supplementary Table 6.** RT-qPCR primers.

| **Gene name** | **Primer** | **Sequence (5’-3’)** |
| --- | --- | --- |
| ***MTHFD2*** | Fwd | AGGACGAATGTGTTTGGATCAG |
|  | Rev | GGAATGCCAGTTCGCTTGATTA |
| ***RPLP0*** | Fwd | TCCTCGTGGAAGTGACATCGT |
|  | Rev | CTGTCTTCCCTGGGCATCA |

Supplementary Materials and Methods:

**Colony formation assay**

Melanoma cells were seeded into 6-well plates at 2,000 cells per well. The next day, belvarafenib, cobimetinib and INK128 (see Supplementary Table 2), or DMSO control were added to the indicated wells. Cells were subsequently cultured for 10 days, during which media was changed and drugs were freshly added every two days. At the end of the assay, cells were fixed with 4% formaldehyde/PBS, stained with 0.5% of crystal violet (Sigma-Aldrich, HT90132) diluted in 70% EtOH and photographed. Colonies were manually quantified using FIJI software (<https://github.com/fiji/fiji>) to assess cell survival and proliferation.

**Western blotting**

Immunoblots were performed as previously described ^28^. Briefly, cells were lysed with RIPA buffer (150mmol/L Tris-HCl, pH=7, 150mmol/L NaCl, 1% NP-40, 1% sodium deoxycholate, 0.1% SDS) supplemented with protease and phosphatase inhibitors (Roche). Equal amounts of protein samples were loaded, separated on 10% or 12% SDS-PAGE gels, transferred to nitrocellulose membranes, and probed with corresponding antibodies. Detailed antibody information is listed in Supplementary Table 4.

**Flow cytometry-based assays**

Melanoma cells were seeded into 6-well plates at 100,000 cells per well. Following indicated treatments, cells were trypsinized, centrifuged at 240g for 5 min, and washed twice in PBS. For apoptosis detection of non-fixed cells, Alexa Fluor™ 647-Annexin V (Invitrogen™, A23204) and Propidium Iodide (PI) Staining Solution (BD Biosciences, 556463) were diluted in 1×binding buffer (BD Biosciences, 556454), and subsequently mixed with cells following the manufacturer’s instructions. All flow cytometry experiments were conducted on the FACSCanto (BD Biosciences).

**RNA sequencing analyses**

Sequencing libraries were generated using NEBNext® UltraTM RNA Library Prep Kit for Illumina® (NEB, USA) following manufacturer’s recommendations. The library was sequenced using the Illumina NovaSeq 6000 sequencing platform to generate raw reads. Then, nf-core/rnaseq pipeline v3.8.1 ^29^ was used to perform quality control, trimming and alignment on raw paired-end fastq reads, followed by reference genome-guided transcriptome assembly and gene expression quantification. Differentially expressed genes (DEGs) were identified by DESeq2 ^30^ with cut-off values of a log2|fold-change| > 1 and a p-adjust < 0.05. ClusterProfiler ^31^ was used to perform functional enrichment analysis and the potential genes in the identified modules were analyzed based on gene ontology (GO) categories.

**RNA interference**

siRNAs were transfected into cells using Lipofectamine™ RNAiMAX Transfection Reagent (Invitrogen, 13778) following the manufacturer’s instructions. Media were changed the next day after cells were incubated with siRNAs for 18 hours. All cells were harvested between 48h-96h after siRNA transfection. All siRNA sequences are listed in Supplementary Table 5.

**Immunofluorescence microscopy**

WM3406 and MeWo cells were grown on glass coverslips and fixed with 4% (w/v) paraformaldehyde (PFA) in PBS for 15 min at room temperature. PFA-fixed cells were permeabilized with 0.2% (v/v) Triton X-100 in PBS for 20 min at room temperature. Cells were then incubated with blocking buffer (5% bovine serum albumin, and 0.5% Triton X-100 in PBS) for 1 hour at room temperature and then incubated with the γH2AX primary antibodies overnight at 4 °C. After three washes with PBS, cells were incubated for 1 hour at room temperature with the secondary antibodies (Invitrogen, A21206). Cells were then washed with PBS, followed by incubation with 1 μg/ml DAPI nuclear stain for 15 minutes. Cells were washed and mounted to a glass slide using ProLong gold mounting media (Invitrogen, P36930). Slides were stored in the dark until fluorescence images were taken. Stacking and coloring of images was performed using FIJI software. Each quantification was done on at least three biological replicates and at least 3 ROIs per biological replicate.

**Quantitative real-time PCR**

Cultured cells were pelleted, and RNA was prepared using the E.Z.N.A. total RNA isolation kit (OMEGA Bio-Tek). RNA concentrations were then quantified using a NanoDrop spectrophotometer (ThermoFisher Scientific) and cDNA was prepared from 1mg of total RNA using iScript cDNA Synthesis Kit (Bio-Rad). Target genes were quantified using the Applied Biosystems 7500 Fast Real-Time PCR System with SYBR Green real-time PCR master mix (Applied Biosystems). The housekeeping genes *RPLP0* was used for each assay. Primers used for qPCR are listed in Supplementary Table 6.

**Immunohistochemistry (IHC)**

Staining of mouse samples was performed as previously described ^1^. Briefly, formalin-fixed, paraffin-embedded tumor sections were stained with indicated antibodies (Supplementary Table 3), followed by a standard magenta red detection protocol ^2^ (Agilent Technologies, GV92511-2). Hematoxylin-counterstained slides were mounted with coverslips. Slides were scanned on AxioScan (ZEISS) and positive staining quantified using QuPath v0.5.1.

**Access and re-analysis of previously published datasets**

Processed count matrices of spatial transcriptomics (Visium) from NRAS^Q61K/°^;Ink4a^−/−^ melanoma mouse model were downloaded from the website of Dr. Jean-Christophe Marine lab (<https://marinelab.sites.vib.be/en>) ^3^. In brief, spots were retained when nFeature_Spatial > 1,000 and percent.mt < 5 and expression data were normalized using SCTransform (Seurat, v.5.0.2) ^4^. To determine the MAPK pathway activity, we used the MPAS score based on the MAPK signaling characteristic gene signatures as previously described ^5^. To determine the mTOR pathway activity, we used Gene Set Variation Analysis (GSVA) ^6^ to perform functional enrichment analysis based on HALLMARK categories. The ISR score was also calculated based on the Z-score of stress response regulators ^7^.

**Tumor dissociation and culture**

MaNRAS1007 and WM3406 melanomas in control group or belvarafenib plus cobimetinib resistant group were resected. Tumors were minced and digested in collagenase A to obtain single-cell suspension. MaNRAS1007 cells were cultured in Ham’s F12 containing 1% FBS, while WM3406 cells were cultured in RPMI containing 1% FBS and 1 x GlutaMAX.

**Reference**

1 Huang, F. *et al.* Inhibiting the MNK1/2-eIF4E axis impairs melanoma phenotype switching and potentiates antitumor immune responses. *J Clin Invest* **131**, doi:10.1172/JCI140752 (2021).

2 Petersen, K. H., Lohse, J. & Ramsgaard, L. Automated sequential chromogenic IHC double staining with two HRP substrates. *PLoS One* **13**, e0207867, doi:10.1371/journal.pone.0207867 (2018).

3 Karras, P. *et al.* A cellular hierarchy in melanoma uncouples growth and metastasis. *Nature* **610**, 190-198, doi:10.1038/s41586-022-05242-7 (2022).

4 Hao, Y. *et al.* Dictionary learning for integrative, multimodal and scalable single-cell analysis. *Nat Biotechnol* **42**, 293-304, doi:10.1038/s41587-023-01767-y (2024).

5 Wagle, M. C. *et al.* A transcriptional MAPK Pathway Activity Score (MPAS) is a clinically relevant biomarker in multiple cancer types. *NPJ Precis Oncol* **2**, 7, doi:10.1038/s41698-018-0051-4 (2018).

6 Hanzelmann, S., Castelo, R. & Guinney, J. GSVA: gene set variation analysis for microarray and RNA-seq data. *BMC Bioinformatics* **14**, 7, doi:10.1186/1471-2105-14-7 (2013).

7 Neill, G. & Masson, G. R. A stay of execution: ATF4 regulation and potential outcomes for the integrated stress response. *Front Mol Neurosci* **16**, 1112253, doi:10.3389/fnmol.2023.1112253 (2023).
