# Supplementary figures and images for: mTOR inhibition enhances the antitumor efficacy of pan-RAF-MEK blockade by inhibiting the ATF4-MTHFD2 pathway
Source: Cell Death Dis. 2026 May 6;17(1):600. doi: 10.1038/s41419-026-08836-5 (PMC13315934; doi:10.1038/s41419-026-08836-5)

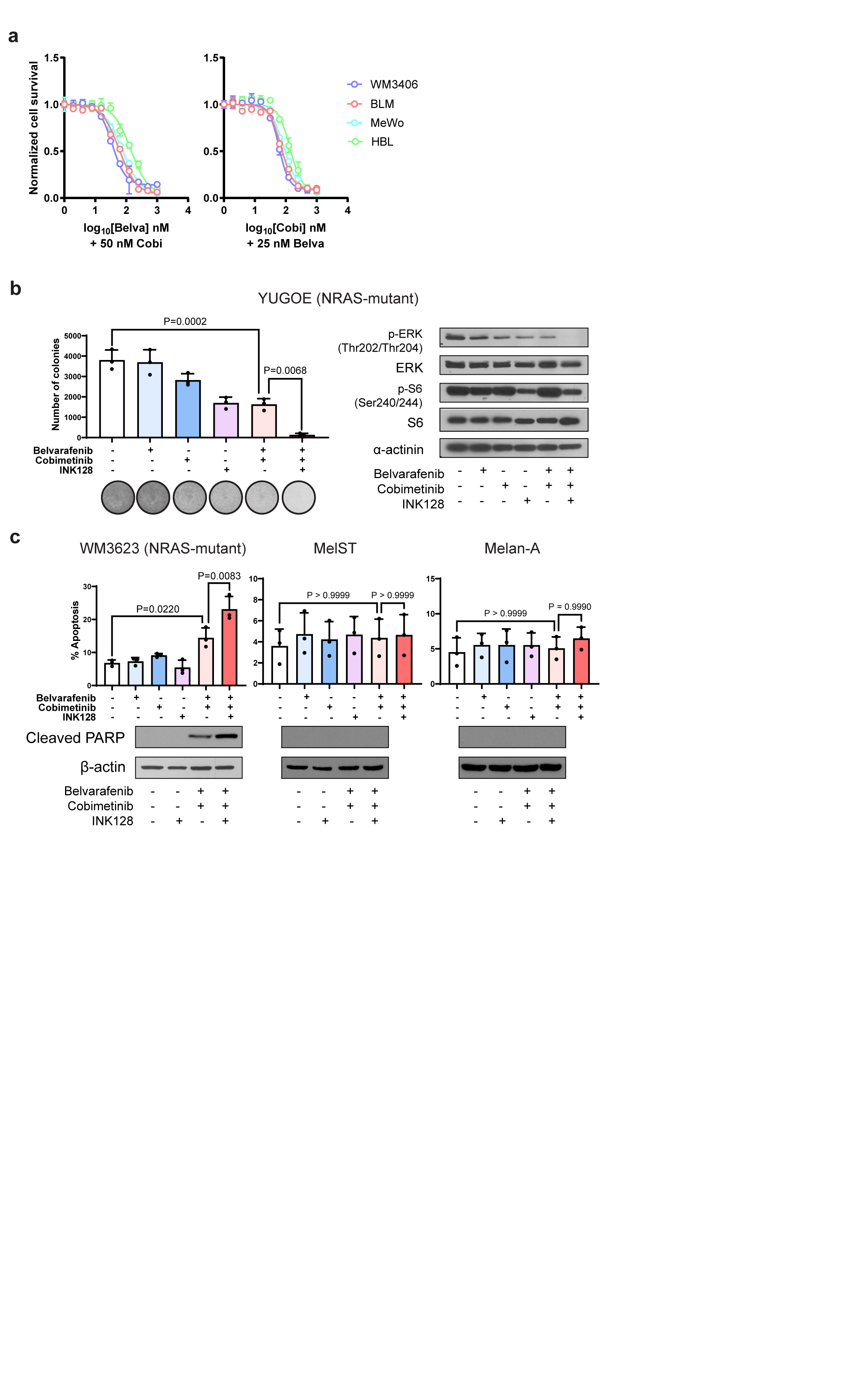

Supplement: Supplementary file 2 — Figure S1 [file 41419_2026_8836_MOESM2_ESM.tif]

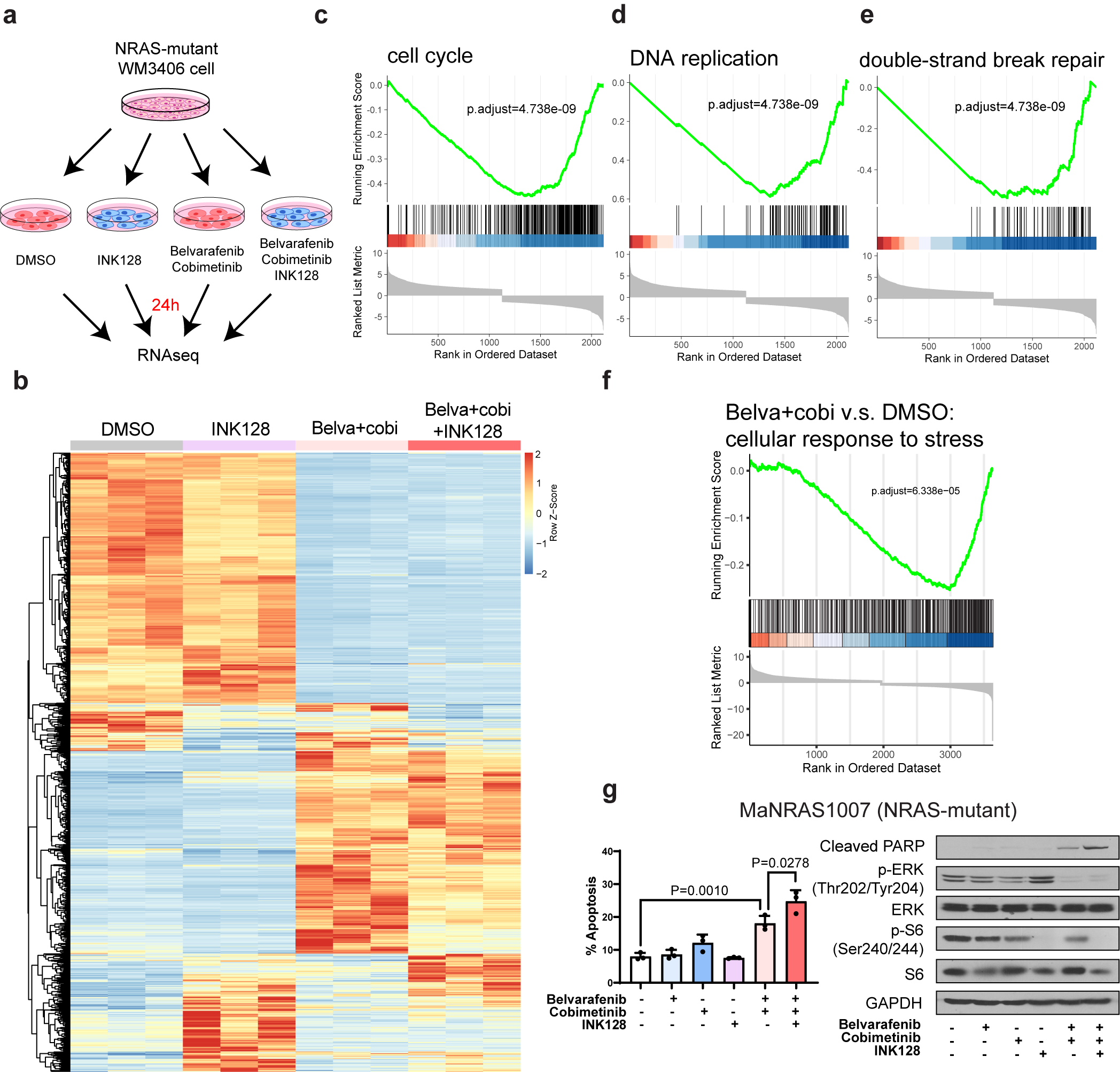

Supplement: Supplementary file 3 — Figure S2 [file 41419_2026_8836_MOESM3_ESM.tif]

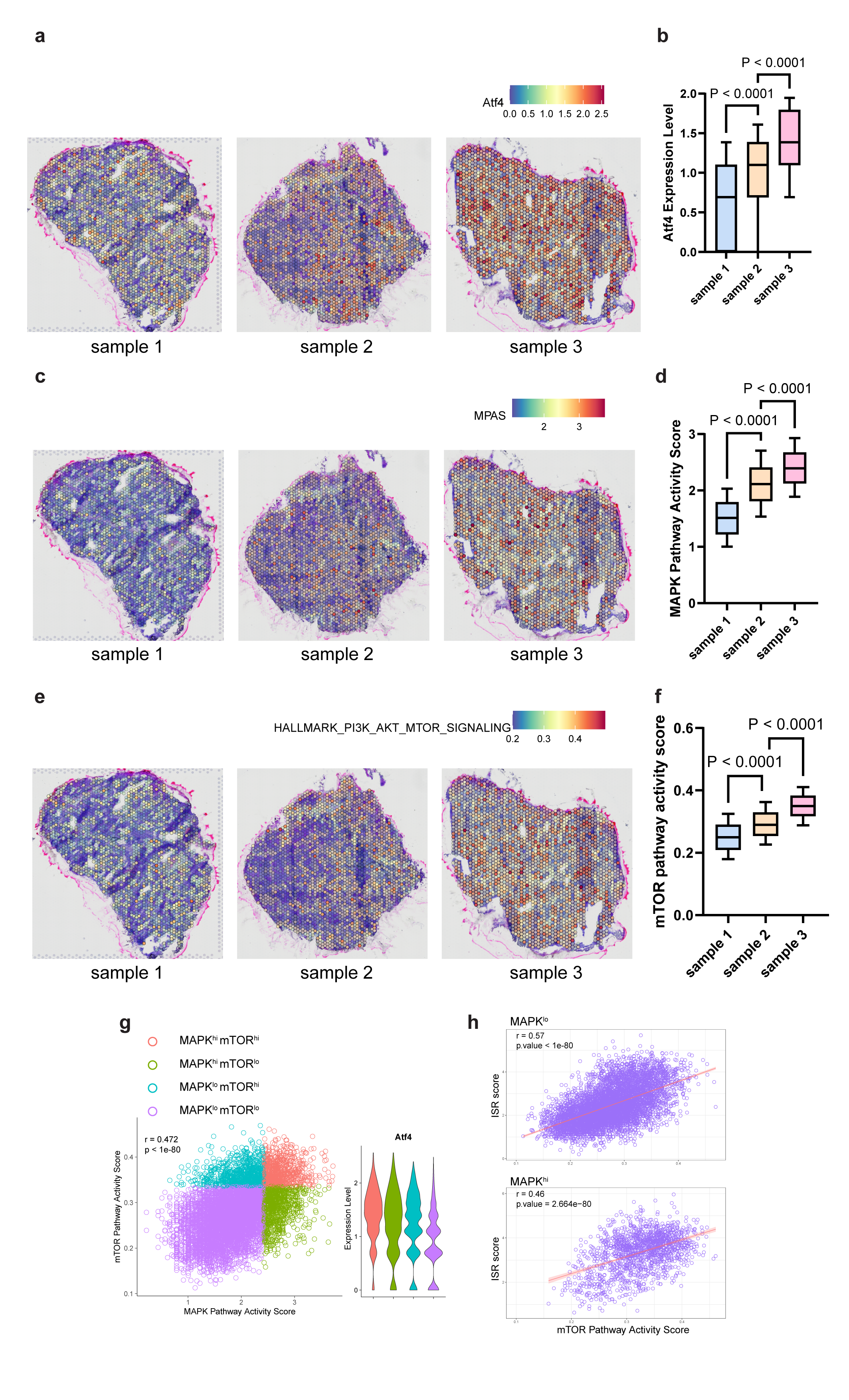

Supplement: Supplementary file 4 — Figure S3 [file 41419_2026_8836_MOESM4_ESM.tif]

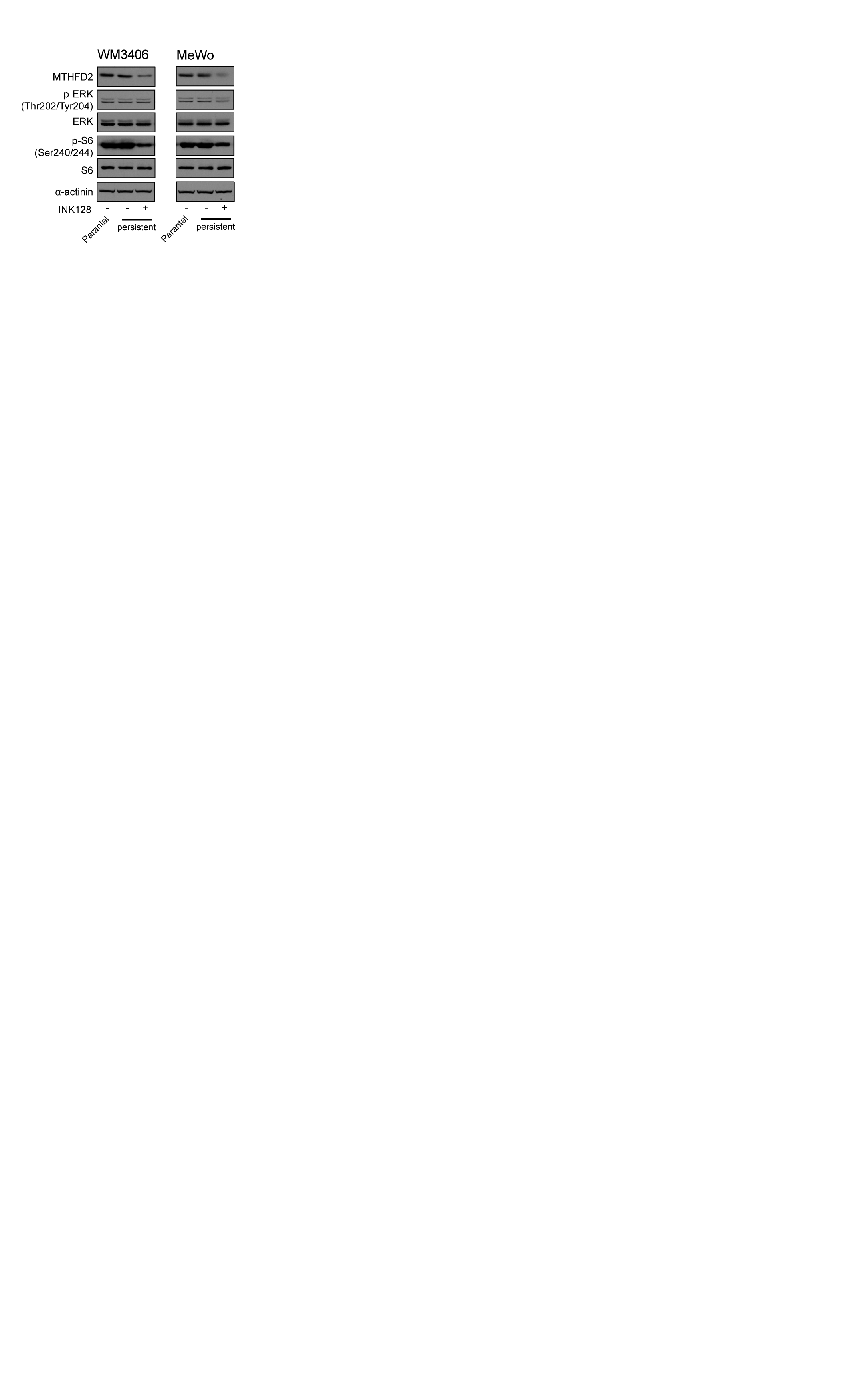

Supplement: Supplementary file 5 — Figure S4 [file 41419_2026_8836_MOESM5_ESM.tif]

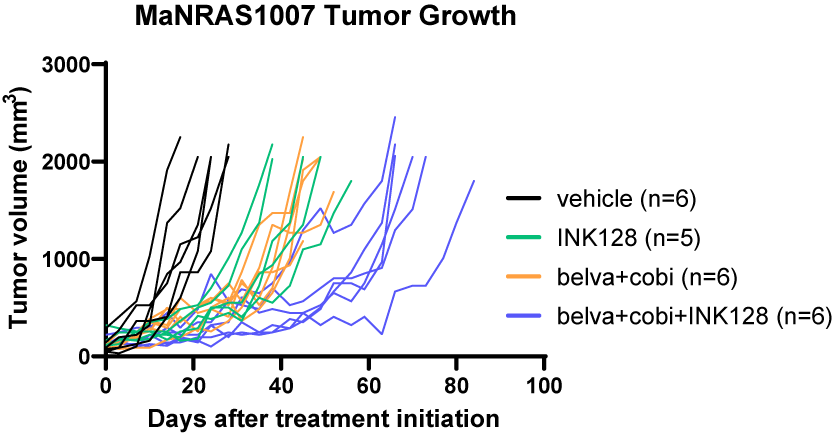

Supplement: Supplementary file 6 — Figure S5 [file 41419_2026_8836_MOESM6_ESM.tif]
