# Supplementary material for: mTOR inhibition enhances the antitumor efficacy of pan-RAF-MEK blockade by inhibiting the ATF4-MTHFD2 pathway
Source: Cell Death Dis. 2026 May 6;17(1):600. doi: 10.1038/s41419-026-08836-5 (PMC13315934; doi:10.1038/s41419-026-08836-5)

Unedited gel for Figure 1b

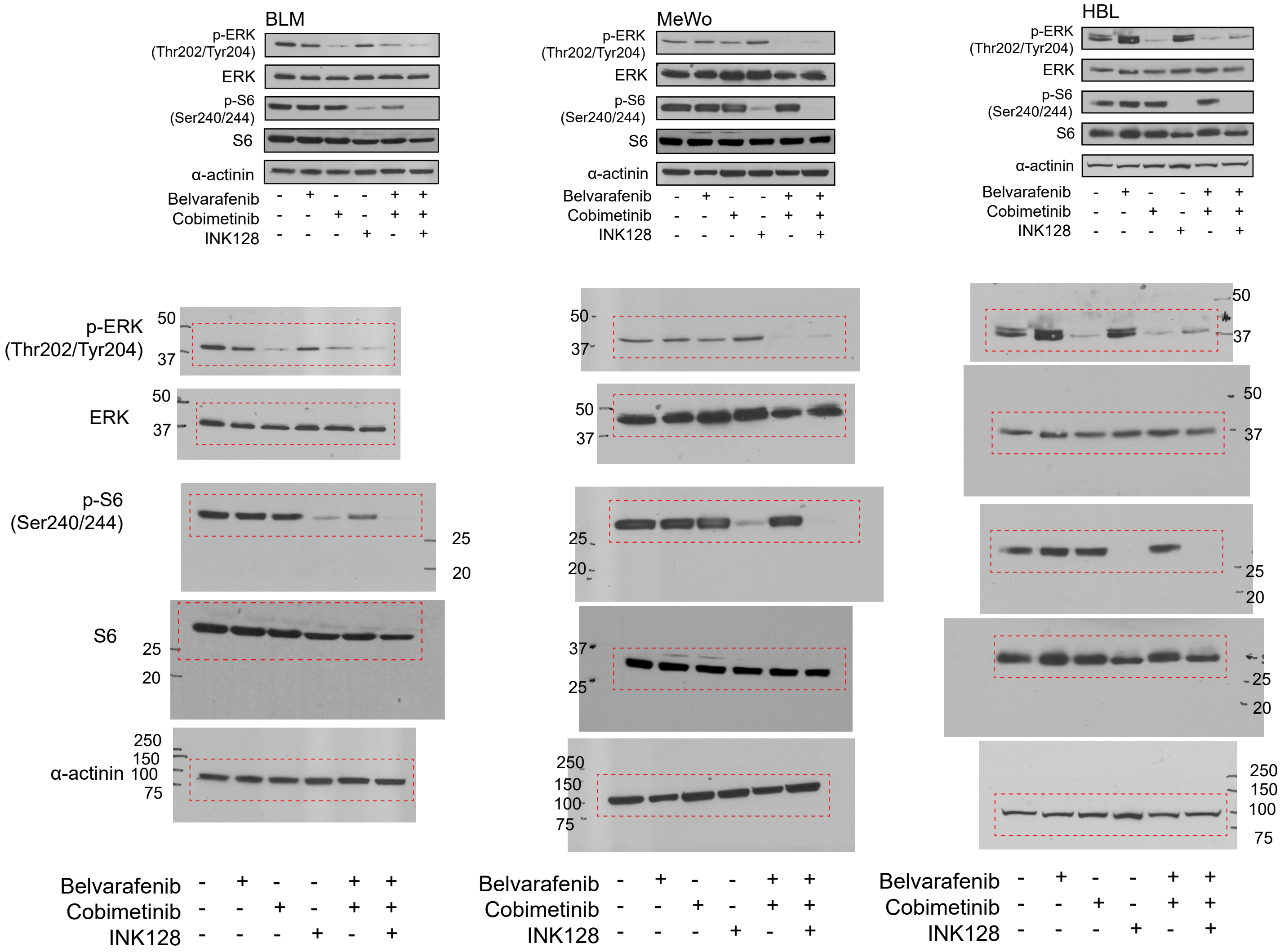

Unedited gel for Figure 1c

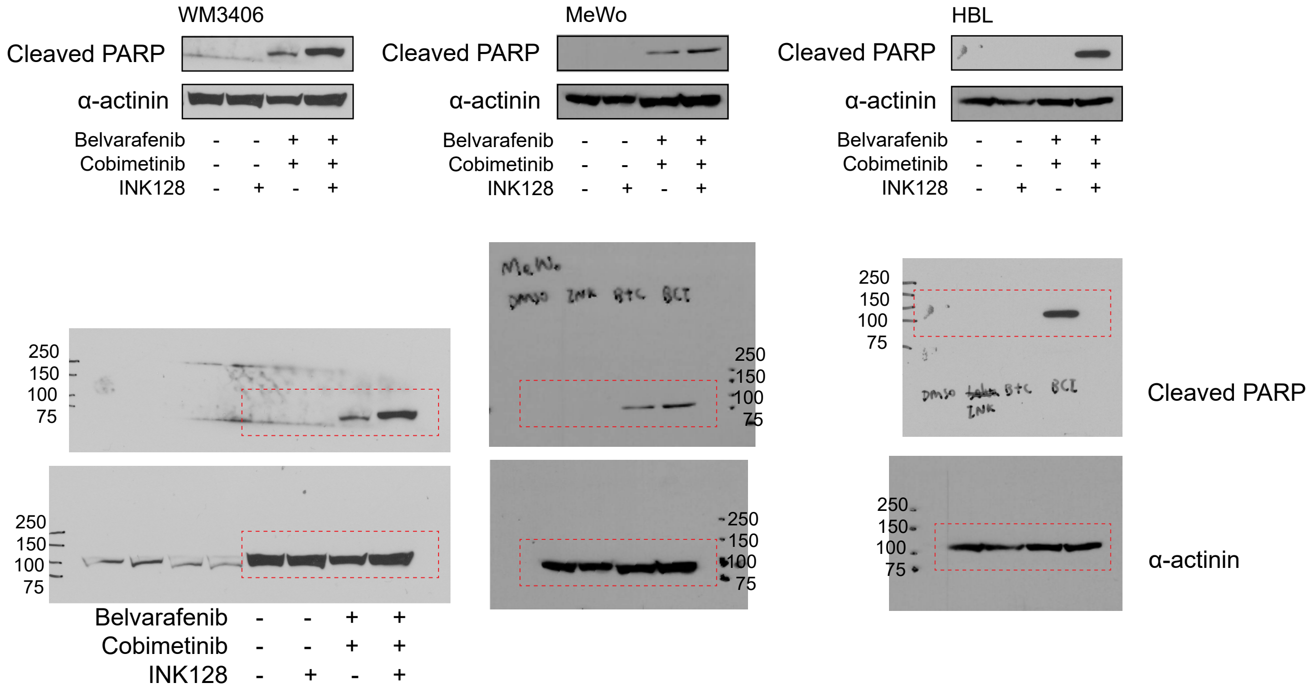

Unedited gel for Figure 3a

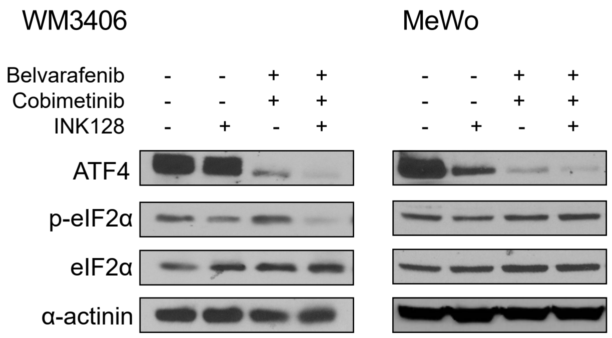

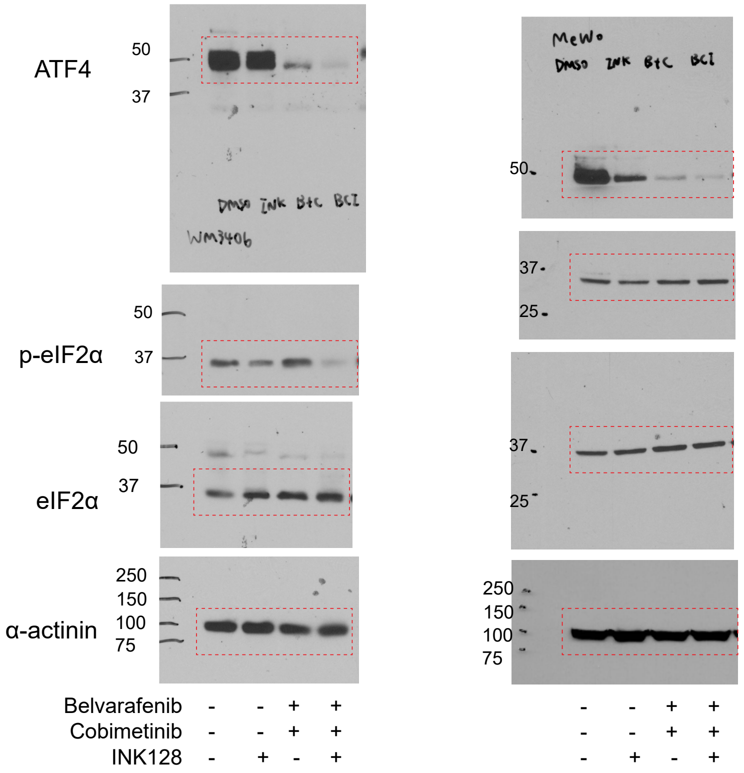

Unedited gel for Figure 3b

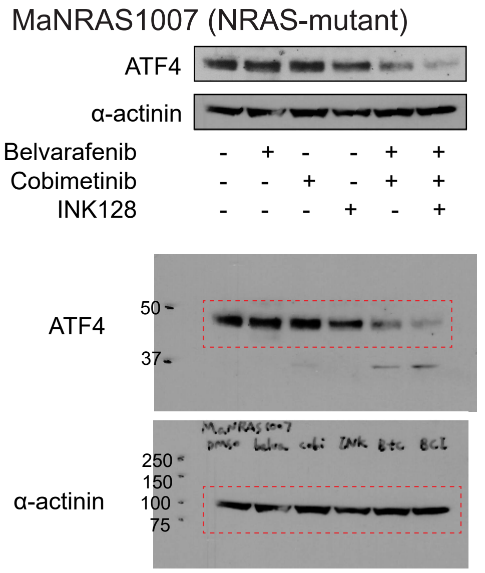

Unedited gel for Figure 3d

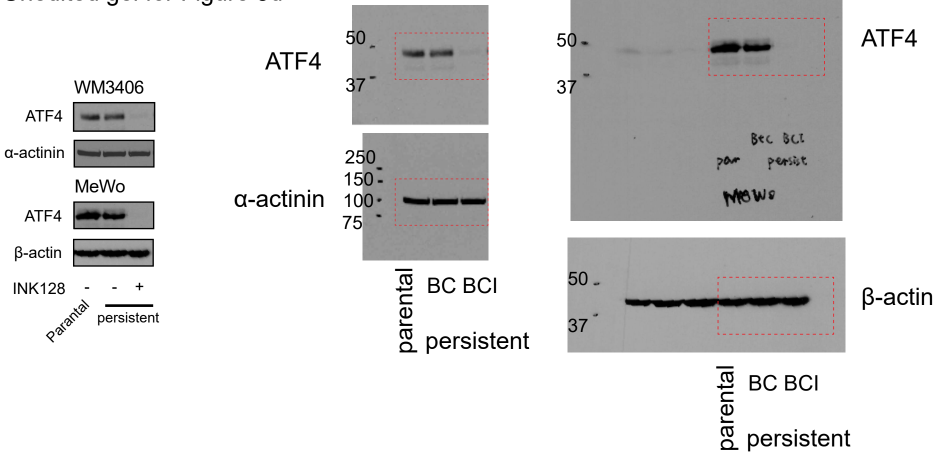

Unedited gel for Figure 3f

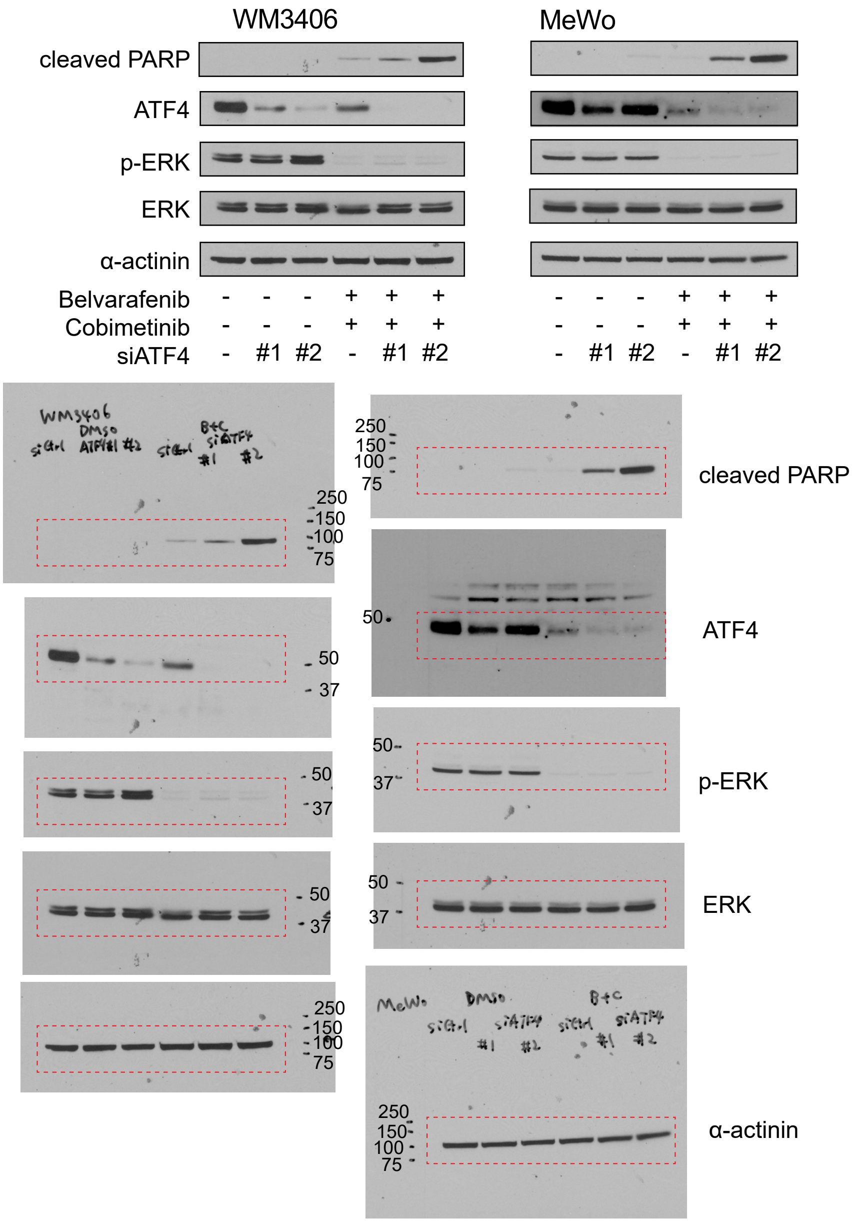

Unedited gel for Figure 3g

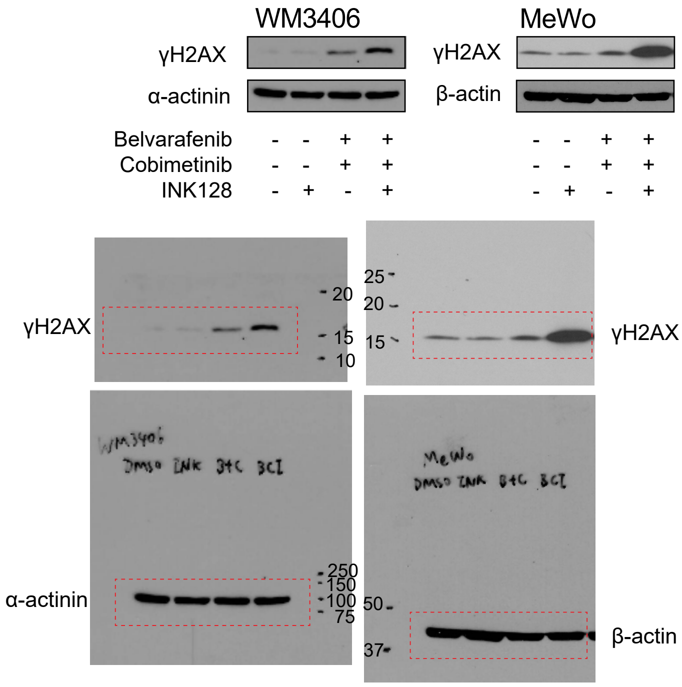

Unedited gel for Figure 3h

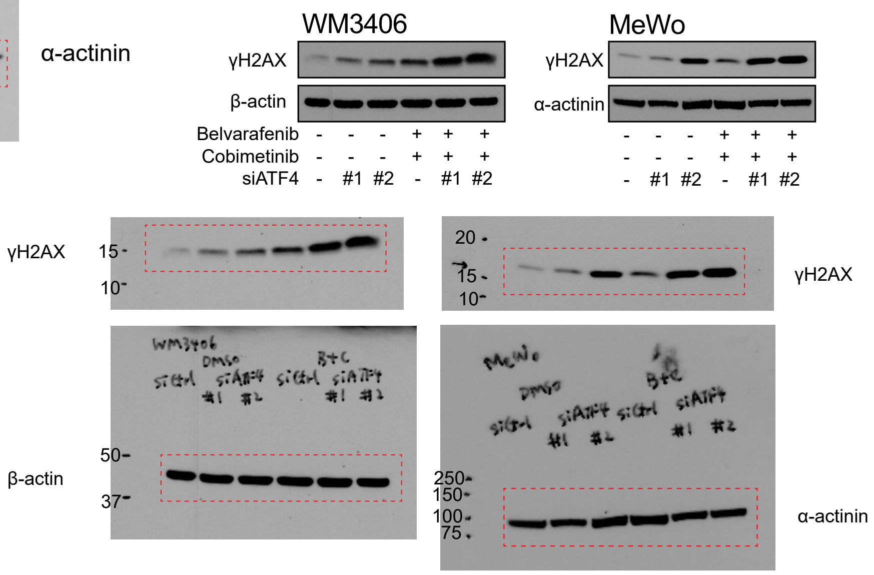

Unedited gel for Figure 4d

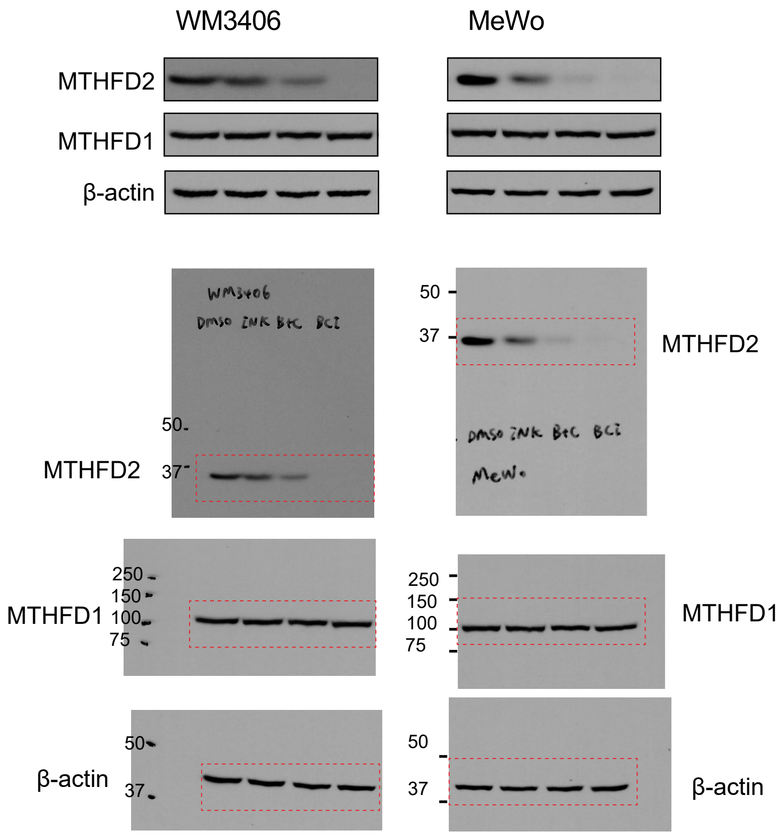

Unedited gel for Figure 4e

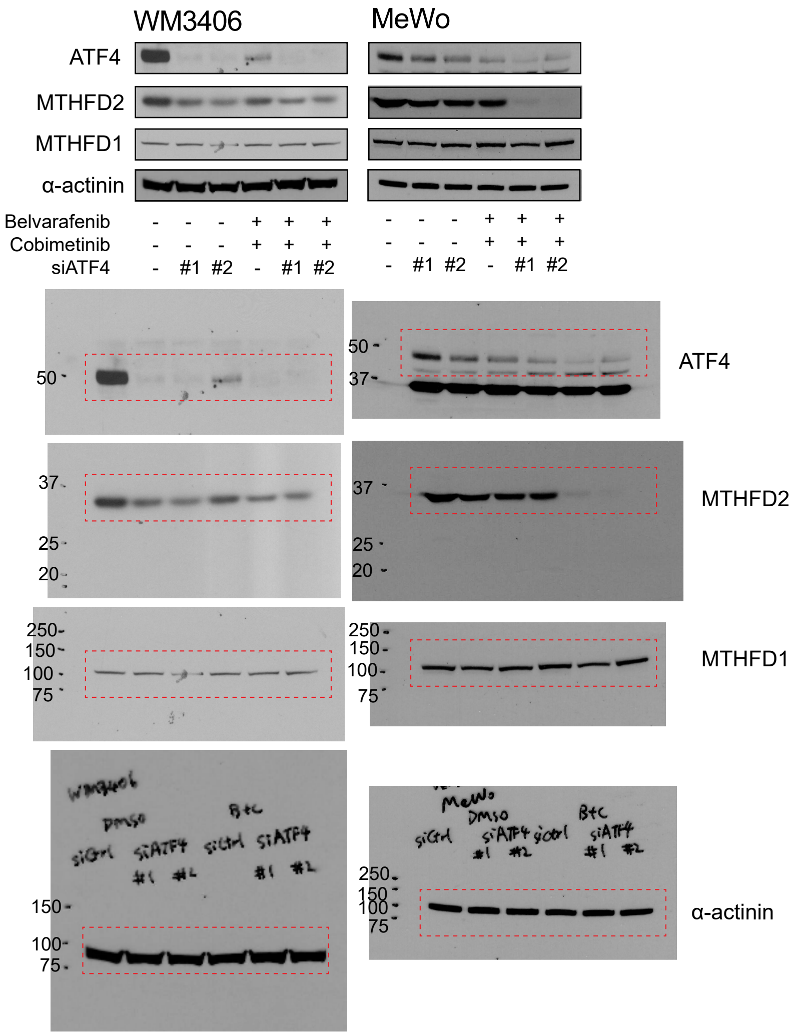

Unedited gel for Figure 5a

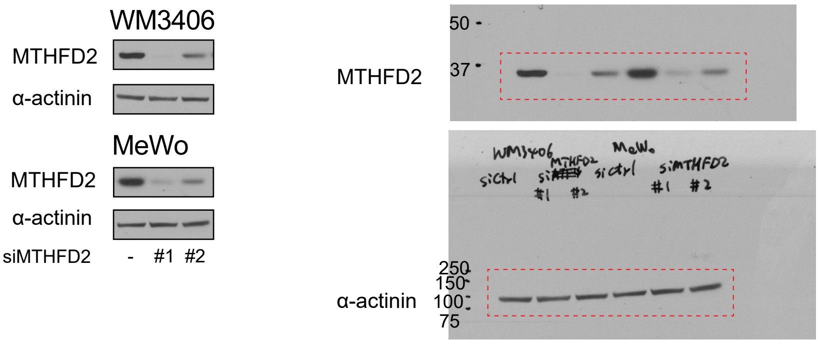

Unedited gel for Figure 5e

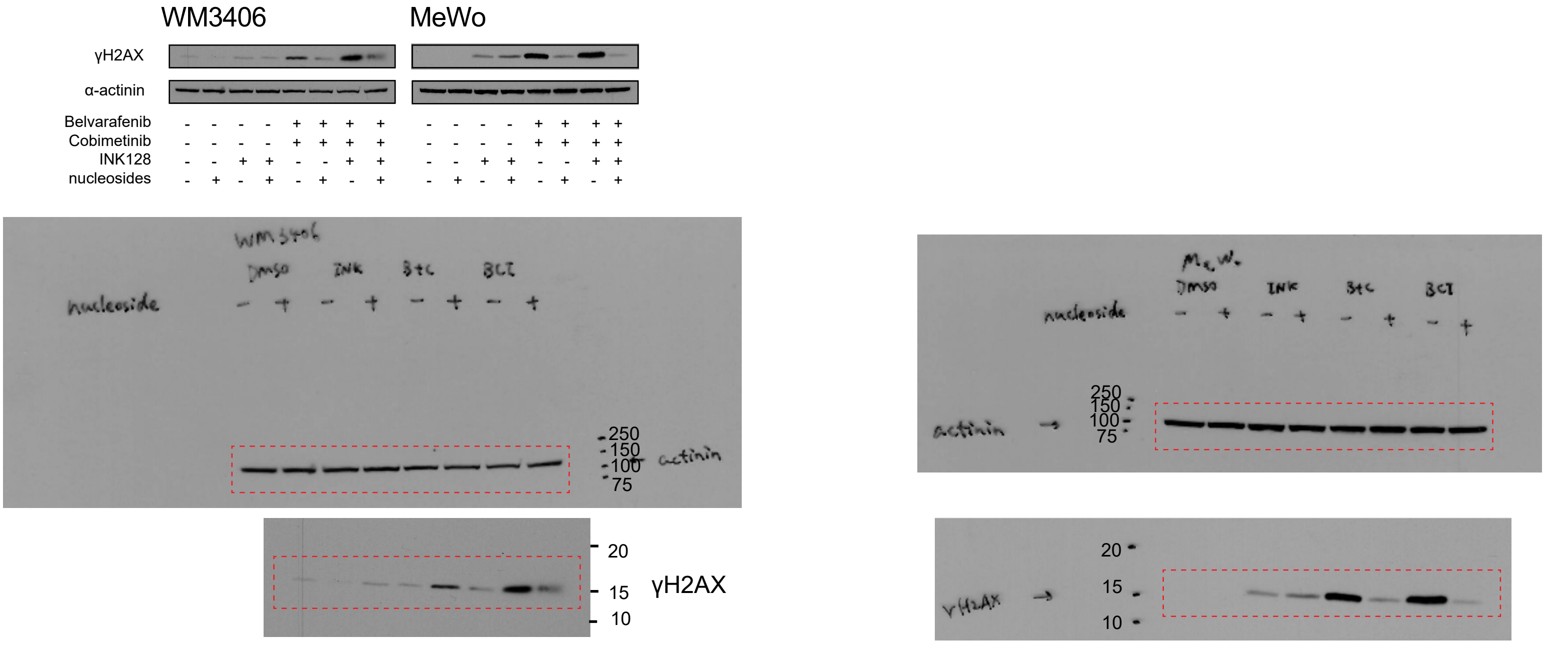

Unedited gel for Figure 7f

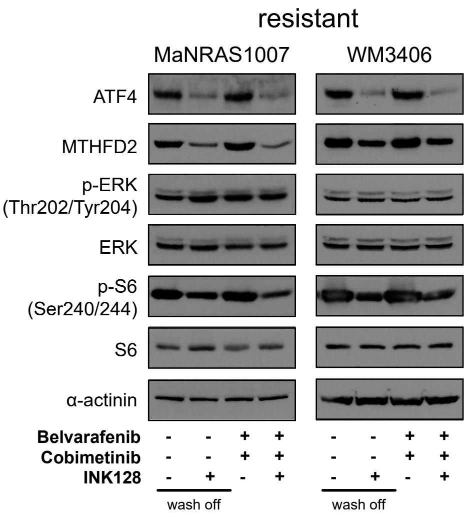

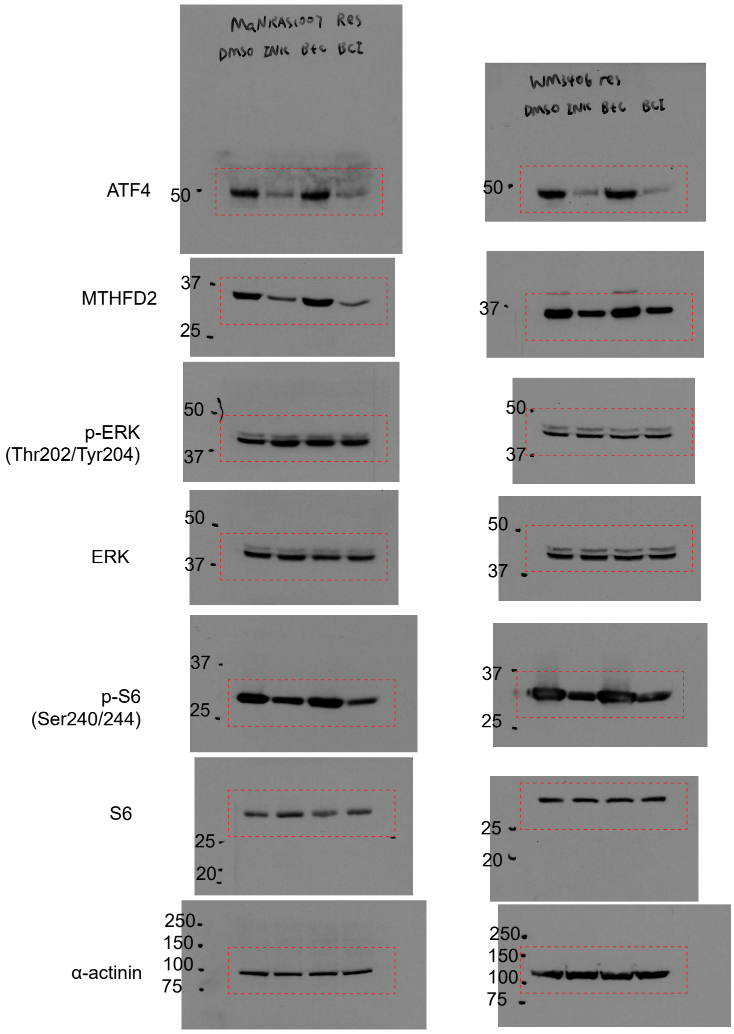

Unedited gel for Figure S1

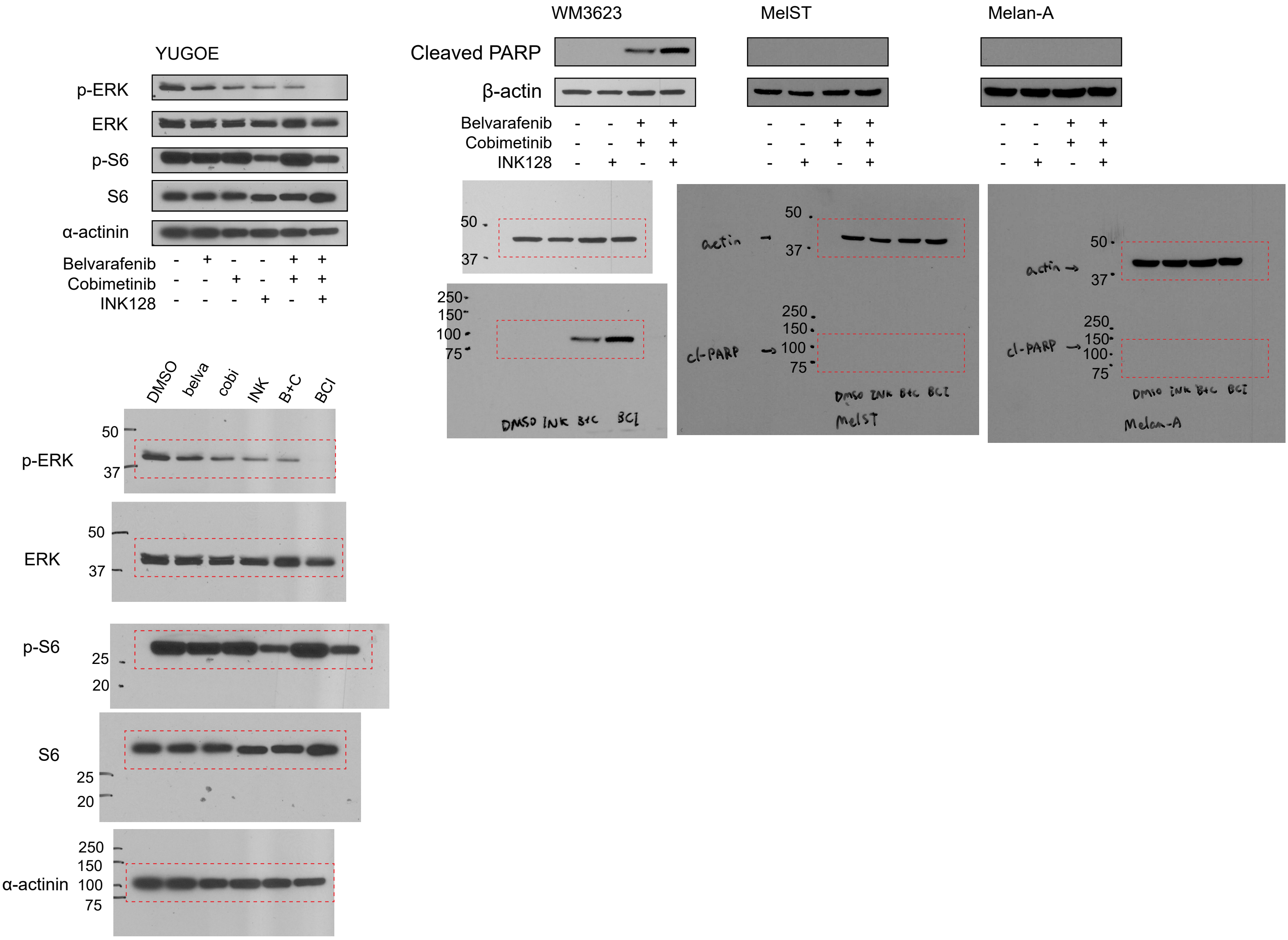

Unedited gel for Figure S2g

MaNRAS1007 (NRAS-mutant)

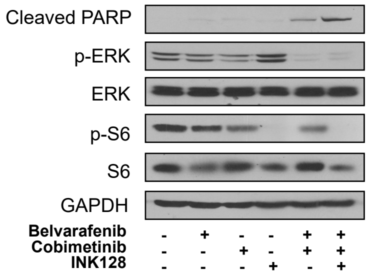

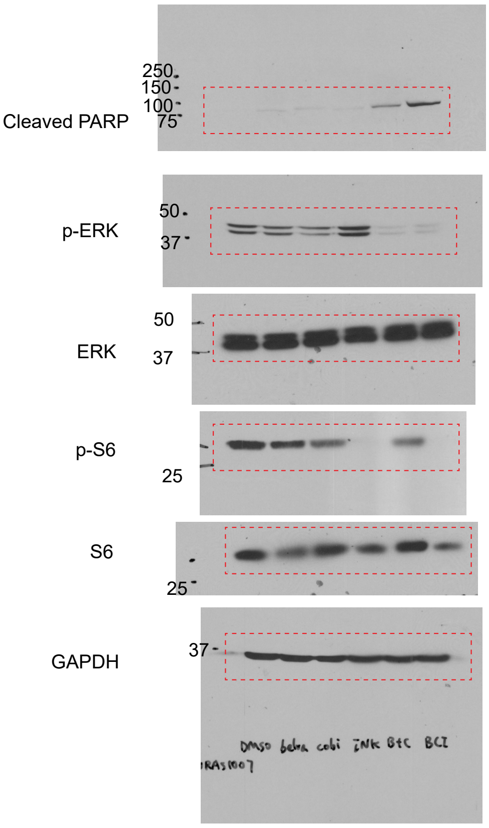

Unedited gel for Figure S4

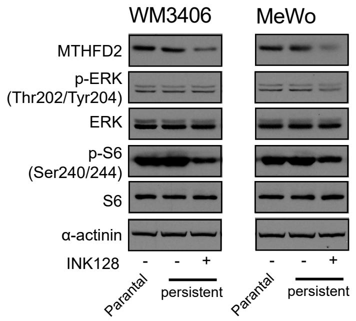

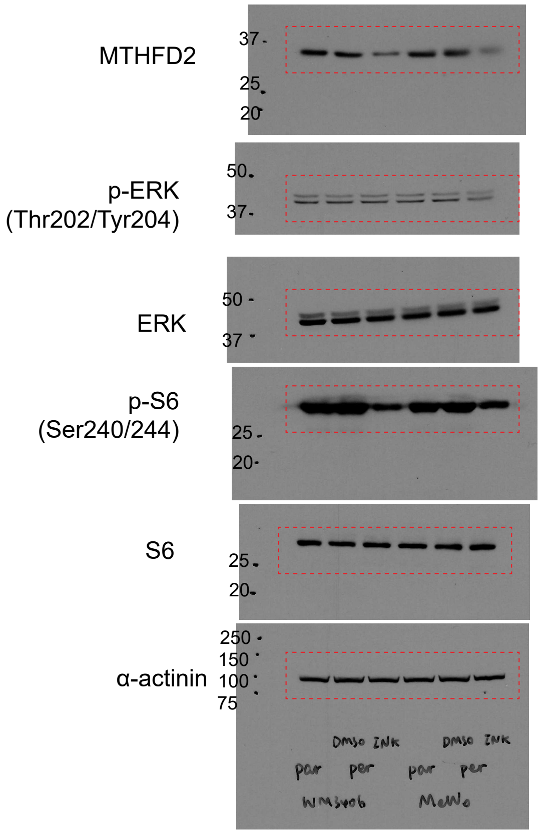

Supplement: Supplementary file 8 — Original Western blots [file 41419_2026_8836_MOESM8_ESM.pdf]
